# Thermally-induced reversible structural isomerization in colloidal semiconductor CdS magic-size clusters

**DOI:** 10.1038/s41467-018-04842-0

**Published:** 2018-06-27

**Authors:** Baowei Zhang, Tingting Zhu, Mingyang Ou, Nelson Rowell, Hongsong Fan, Jiantao Han, Lei Tan, Martin T. Dove, Yang Ren, Xiaobing Zuo, Shuo Han, Jianrong Zeng, Kui Yu

**Affiliations:** 1 Institute of Atomic and Molecular Physics, Sichuan University, 610065 Chengdu, PR China; 2 School of Materials Science and Engineering, Huazhong University of Science & Technology, 430074 Wuhan, PR China; 3 National Research Council of Canada, Ottawa, Ontario K1A 0R6 Canada; 4 Engineering Research Center in Biomaterials, Sichuan University, 610065 Chengdu, PR China; 5 School of Physics and Astronomy, Queen Mary University of London, London, E1 4NS UK; 6 School of Physical Science and Technology, Sichuan University, 610065 Chengdu, PR China; 7 X-ray Science Division, Advanced Photon Source, Argonne National Laboratory, Lemont, IL 60439 USA; 8 Shanghai Synchrotron Radiation Facility, Shanghai Institute of Applied Physics, Chinese Academy of Sciences, 201204 Shanghai, PR China; 9 School of Chemical Engineering, Sichuan University, 610065 Chengdu, PR China

## Abstract

Structural isomerism of colloidal semiconductor nanocrystals has been largely unexplored. Here, we report one pair of structural isomers identified for colloidal nanocrystals which exhibit thermally-induced reversible transformations behaving like molecular isomerization. The two isomers are CdS magic-size clusters with sharp absorption peaks at 311 and 322 nm. They have identical cluster masses, but slightly different structures. Furthermore, their interconversions follow first-order unimolecular reaction kinetics. We anticipate that such isomeric kinetics are applicable to a variety of small-size functional nanomaterials, and that the methodology developed for our kinetic study will be helpful to investigate and exploit solid–solid transformations in other semiconductor nanocrystals. The findings on structural isomerism should stimulate attention toward advanced design and synthesis of functional nanomaterials enabled by structural transformations.

## Abstract (web summary)

Few structural isomers of colloids, with identical masses but different structures, have been identified. Here, the authors observe an interesting example of structural isomerism in a pair of semiconductor magic-size clusters, which reversibly transform between one another with first-order unimolecular reaction kinetics.

## Introduction

Solid–solid transformations take place between two solid states^1–19^, with growing evidence for their applicability to colloidal semiconductor nanocrystals (NCs)^1–4^. Such transformations are known to play critical and determinant roles in the physical, optical, magnetic, and electronic properties of macroscopic materials, and we anticipate the same will be true for nanoscale materials. Solid–solid transformations can be initiated by changes in experimental conditions including temperature and pressure.

The kinetics of transformations^1–8^, such as the pressure-induced four-coordination to six-coordination transformations of semiconductor CdSe and CdS^1–5^ and hydriding transformations of palladium NCs^6^, have been reported to be affected significantly by material sizes. In the bulk, a phase transformation begins with nucleation in a parent phase followed by growth. However, when the size of a NC (consisting of 10^2^–10^3^ atoms) is comparable to that of a critical nucleus (estimated for the solid–solid transformations in the bulk)^1,6,20^, a coherent deformation of the entire nanocrystal takes place in a single step. As illustrated in Supplementary Table 1, such a transformation, which features a single nucleation event without further growth taking place within a confined nanoscale range, follows a first-order reaction kinetic behavior^1,4^.

A colloidal semiconductor magic-size cluster (MSC) usually consists of a relatively small number of atoms, being smaller than 3 nm in size^21–26^. From a single wet chemistry batch, a certain degree of size distribution is unavoidable for a conventional semiconductor NC product, except for MSCs. Each type of MSC has a very tight size distribution, exhibiting a much sharper bandgap absorption than the corresponding conventional quantum dots (also called regular quantum dots (RQDs)). The very narrow optical bandwidth associated with single-ensemble MSCs has been attributed to their enhanced structural stability, hence the adjective “magic” is used. For thiolated gold MSCs (consisting of 144 Au atoms) and for clusters (consisting of 38 Au atoms)^12,13^, polymorphism and irreversible structural isomerism have been reported, respectively. Structural isomerism of colloidal semiconductor MSCs has not yet been reported^21–37^, with the synthesis of suitable single-ensemble MSCs being one of the challenges.

Recently, we reported a two-step synthetic approach for colloidal MSCs in a single-ensemble form without the simultaneous production of other-size NCs^27,28^. We demonstrated that the evolution of CdS MSC-311, referred to as the wavelength in nanometers (nm) of their absorption peak position, from their immediate precursors (IPs) followed first-order unimolecular reaction kinetics. The IPs of MSCs were essentially transparent in optical absorption; during the IP formation, an increase of the absorbance at shorter wavelengths (~290 nm) was observed. During the transformation to MSCs, a continuous increase of the absorbance at 311 nm and a simultaneous decrease of the absorbance at 290 nm were detected, together with a concurrent redshift (larger than 10 nm) of the peak position to 311 nm. We concluded that the continuous redshift was not due to the increase of the mass of NCs, similar to the continuous redshift observed for the crystallization process of PbSe MSCs^37^. The redshift was indicative of a structural transformation leading to the formation of optically visible CdS MSC-311, which was understood to take place via a two-step pathway. The structural transformation from the IP to MSC-311 was attributed to the intramolecular reorganization caused by a proton-mediated ligand exchange^28,38^, and was independent of the concentration of the IP and thus of the degree of supersaturation^28^.

In the current work, we report the thermally-induced reversible structural isomerism between CdS MSC-311 and MSC-322. The two types of MSCs are synthesized in a single-ensemble form. We demonstrate that the MSCs are isomeric with the same cluster mass, but have slightly different structures based on matrix-assisted laser desorption/ionization time-of-flight (MALDI-TOF) mass spectrometry (MS), X-ray total scattering with atomic pair distribution function (PDF) analysis, and small angle X-ray scattering (SAXS). The two types of MSCs interconvert in a reversible fashion via the ready change of temperature. In situ time-resolved optical absorption spectroscopy of the reconstructive solid–solid transitions reveals an isosbestic point around 317 nm, supporting a mechanism of direct interconversions without the involvement of intermediates. Both the forward and reverse transformations follow first-order unimolecular reaction kinetics, similar to what occurs in molecular isomerization^39^. Accordingly, the thermally-induced reversible solid–solid transformations of MSC-311⇔MSC-322 most likely feature single- nucleation events^1,4^. For the MSC-311⇒MSC-322 transformation, an Arrhenius analysis suggests the presence of possible breakage of Cd–S bonds^40^. The present study identifies one pair of structural isomers of semiconductor NCs that exhibits thermally-induced reversible transformations obeying first-order unimolecular reaction kinetics. We anticipate that such single-nucleation events may generally occur with similar kinetic behaviors for structural transformations from IPs to MSCs and among various isomeric MSCs, regardless of the nature of materials and induction methods (such as changes in pressure or temperature)^1–4,27,28^.

## Results

### Synthesis of CdS MSC-311 and MSC-322

The two-step approach to CdS MSCs involved first heating Cd and S precursors at a relatively high temperature (such as 180 °C), and second cooling the resulting mixture to a relatively low temperature and dispersing it into a solvent, such as cyclohexane or toluene^28^. When a reaction mixture was cooled to room temperature, CdS MSC-311 and/or MSC-322 were detected. During the course of our work, we found that the exact temperature employed during the second step was critical for the evolution of the two types of MSCs, which were monitored in situ by optical absorption spectroscopy.

**Fig. 1:**
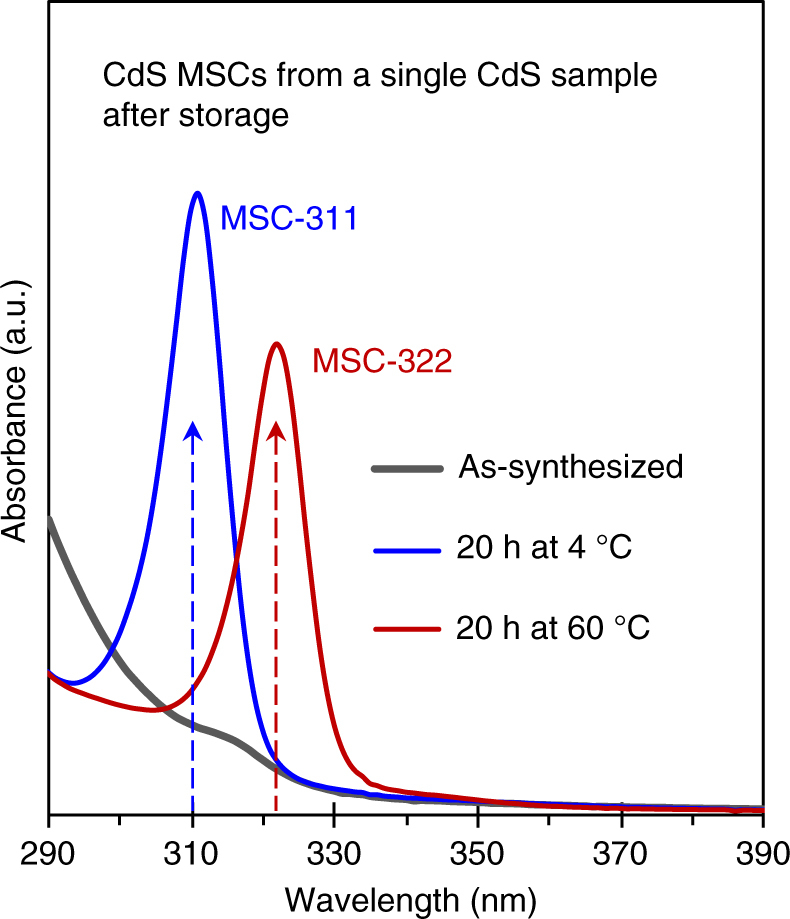
Single-size CdS MSC-311 and MSC-322 prepared from one reaction mixture. Optical absorption spectra of CdS MSC-311 (blue trace) and MSC-322 (red trace), collected from a single preheated sample (~20 mg) (gray trace) after 20 h incubation at 4 and 60 °C, respectively. The dispersion solvent used was cyclohexane (3 mL). To prepare the sample, Cd(OA)_2_ and SODE were mixed at room temperature with a 4 to 1 feed molar ratio and a S concentration of 30 mmol/kg in ODE; the mixture was heated up to 180 °C and was held for 20 min. For the optically transparent IP (gray trace) after stored at 4 °C for 20 h, single-size CdS MSC-311 developed, while at 60 °C for 20 h, single-size CdS MSC-322 evolved

In Fig. 1, we show the absorption spectra of CdS MSC-311 (blue trace), MSC-322 (red trace), and their IPs (gray trace). The two types of MSCs were developed from the same IP but at the two different incubation temperatures. The IP had been prepared in 1-octadecene (ODE) from one mixture of cadmium oleate (Cd(OA)_2_) and SODE with the feed molar ratio of 4 to 1 and a feed S concentration of 30 mmol/kg. The reaction mixture was heated from room temperature to 180 °C where it was held for ~20 min. The as-synthesized IP in cyclohexane seemed to be optically transparent. After the reaction mixture was cooled down, then incubated at 4 °C for 20 h and dispersed in cyclohexane, MSC-311 was detected. When the incubation temperature was 60 °C instead of 4 °C, MSC-322 predominated. In both cases, there were no other-size NCs produced, and the MSC absorption peak was narrow, with a full-width at half-maximum (FWHM) of ~10 nm, similar to what has been reported typically for Cd-based MSCs^27–36^.

It is thus evident that the incubation temperature has a critical influence on the development of MSC-311 and MSC-322 from the as-prepared IP. Over a wider range, we found that a relatively low incubation temperature favored the evolution of MSC-311, while a higher temperature promoted the growth of MSC–322. Supplementary Fig. 1 presents the absorption spectra collected after the reaction mixture was cooled down and stored at six different temperatures, –196, –20, 4, 30, 60, and 80 °C. At –196 °C, there was no apparent development of MSCs of any types; thus, pre-heated reaction mixtures can be kept at this temperature for future usage. At –20 and 4 °C, MSC-311 developed, but the quantity obtained in 20 h was smaller at –20 °C than that at 4 °C. At the higher temperatures, MSC-322 grew, but with a faster rate at 80 °C than at 60 °C or at 30 °C. Supplementary Fig. 2 shows the temporal evolution of MSC-311 at 4 °C and that of MSC-322 at 60 °C, both for storage periods up to 20 h. It seems reasonable that MSC-311 and MSC-322 prefer relatively low and high temperatures to develop, respectively, while the consumption of the IP took place relatively quickly in the first two hours. In previous reports, CdS MSC-311 and/or MSC-322 usually occurred together with CdS RQDs from a single synthetic batch^29–34^. It is interesting to synthesize MSC-311 and MSC-322 from one optically transparent IP sample extracted from one reaction batch^28–34^.

### MALDI-TOF MS and atomic PDF analysis

**Fig. 2:**
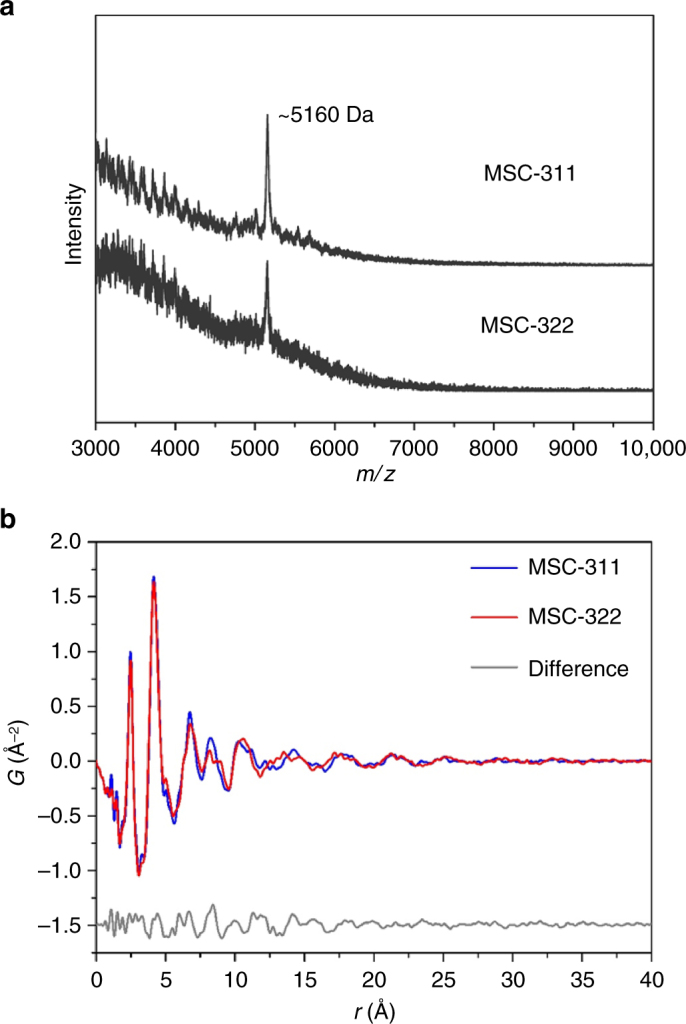
Mass and structural characterization of MSC-311 and MSC-322. **a** MALDI-TOF mass spectra of MSC-311 and MSC-322 with the *m*/*z* range from 3000 to 10,000 Da. The species at ~5160 Da were detected for both the clusters. **b** PDF analysis of MSC-311 (blue trace) and MSC-322 (red trace), together with their intensity difference (gray trace obtained by MSC-311 subtracting MSC-322 with offset). For both the clusters, the first two sharp peaks (at 2.46 and 4.15 Å) are similar, suggesting that the nearest-neighbor Cd–S, Cd–Cd, and S–S distances are similar. The sharp short-range structural correlations indicate intra-cluster distances up to 10–20 Å (beyond which there are no further sharp correlation peaks). Accordingly, the two clusters have similar sizes. Meanwhile, the difference between the two PDF patterns in the range of 5–17 Å (gray trace) provides evidence that the two clusters have different structures

MSC-322 was assumed to have a larger volume, with an empirical estimate of the volume ratio of MSC-322 to MSC-311 of 1.6 based on their absorption peak positions^34^. And MSC-311 was reported to form a part of MSC-322^31^. To explore the cluster masses, we used MALDI-TOF MS (Fig. 2a). To compare the cluster structures, we employed PDF analysis of X-ray total scattering (Fig. 2b), in addition to powder X-ray diffraction (XRD) and wide-angle X-ray scattering (WAXS). Strikingly, our results addressed in this section show that MSC-311 and MSC-322 have very similar cluster masses, but are comprised of slightly different structures.

MS has been used to characterize the mass of colloidal NCs^28,41–46^. For minimal fragmentation based on a soft ionization, MALDI uses laser energy to generate charged species from molecules, clusters, and NCs. In the present work, two compounds, *trans*-2-[3-(4-*tert*-Butyl-phenyl)-2-methyl-2-propenylidene] malononitrile (DCTB) and α-Cyano-4-hydroxycinnamic acid (CHCA), were used as our mass-study matrices. Figure 2a and Supplementary Fig. 3 show the MALDI-TOF mass spectra collected from MSC-311 and MSC-322 dispersions which were mixed with DCTB and CHCA, respectively. The peaks of two MSCs were detected at ~5160 Da in both matrices. Thus, the two clusters have essentially the same mass and, therefore, most probably the same composition. Supplementary Fig. 4 shows thermogravimetric analysis (TGA) of MSC-311 and MSC-322, suggesting similar weight ratios of their organic ligands to inorganic cores.

PDF analysis of X-ray total scattering has been applied to obtain information regarding structures of colloidal NCs^12,26,47^. The details of our PDF study can be found in Supplementary Methods. To obtain MSCs in a powder form, we found that, for the CdS MSCs passivated by conventional oleate ligands, purification resulted in the presence of RQDs in the purified product. Such a coexistence was an obstacle to precise structural characterization. To prevent the formation of RQDs during purification, we used phenylacetic acid^26,47^ to synthesize MSC-311 and MSC-322. For our structural characterization, the MSCs used were passivated by phenyl acetate ligands instead of by conventional oleate ligands. As shown in Supplementary Fig. 5, the two types of MSCs passivated by phenyl acetate ligands displayed no significant shift of their optical absorption peaks after purification, and there was no notable indication of the presence of RQDs.

Figure 2b shows the PDF analysis of MSC-311 (blue trace), MSC-322 (red trace), and their difference (gray trace). For the two types of MSCs, their first (at ~2.46 Å) and second (at ~4.15 Å) peaks overlap quite closely with each other. As illustrated by Supplementary Figs. 6–8, the first peak represents the nearest-neighbor Cd–S correlation, while the second peak represents the nearest-neighbor Cd–Cd and S–S correlations. The similarity in the first two peak positions indicates a very similar local structure (including the average coordination number) for these two clusters. Meanwhile, the intra-cluster correlations for both MSC-311 and MSC-322 seem to exist within a similar distance, which can be estimated to be between 1 and 2 nm, according to the end of the sharp correlation peaks. Thus, the two clusters have similar sizes of 1–2 nm. Crucially, there are some differences between the PDFs of MSC-311 and MSC-322, most notably with regard to the fourth peak at a distance of ~8 Å. This fourth peak appears to be one peak for MSC-311, while two separate peaks for MSC-322. The difference between the PDFs of MSC-311 and MSC-322 is represented by the gray trace in Fig. 2b, which can be seen mainly in the range of 0.5–1.7 nm. The PDF analysis indicates that the structures of the two types of MSCs have some differences but local structures (corresponding to the first and second peaks) are similar. To endorse such a conclusion, synchrotron-based X-ray total scattering experiments with PDF analyses were performed, together with X-ray absorption fine structure (XAFS) and SAXS. These three synchrotron-based experiments were performed on the same two cluster samples (shown by Supplementary Figs. 9–11, respectively); more discussions on size and shape are addressed in the captions of Supplementary Figs. 6–11.

Further evidence for the structural difference between the two types of MSCs has been obtained from, WAXS and XRD, as shown by Supplementary Figs. 12,13, respectively. We would like to point out that TEM, as shown by Supplementary Fig. 14, is not able to deliver the accurate size information for small-size colloidal NCs^32,43^. Our mass and structural investigation provided strong experimental evidence that MSC-311 and MSC-322 have the same cluster mass but different structures. Thus, they probably are one pair of structural isomers identified for semiconductor NCs. We believe that such identification with outstanding fundamental implications will generate experimental interests on the exploration of isomeric MSCs and motivate theoretical efforts for an in-depth understanding of the dimension of structures on semiconductor bandgap engineering.

### Thermally-induced reversible transformations between CdS MSC-311 and MSC-322

It has been generally accepted that colloidal semiconductor MSCs disappear and/or grow into larger MSCs and/or RQDs upon heating^21,24–37^. For example, CdS MSC-311 was reported to grow into MSC-322 with a supply of thermal energy^31,32^. In this work, we monitor their thermally-induced reversible transformations and to explore transformation kinetics, with in situ time-resolved optical absorption spectroscopy.

**Fig. 3:**
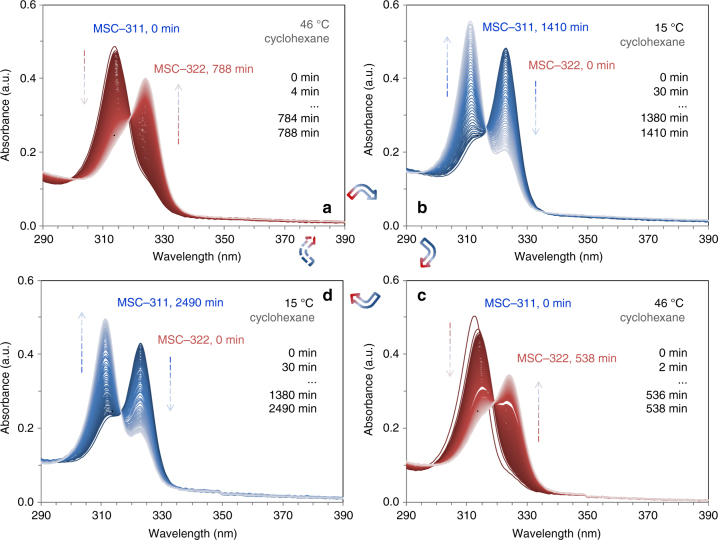
Thermally-induced reversible transformations between MSC-311 and MSC-322. In situ time-resolved optical absorption spectra collected from one sample (9.4 mg) in toluene (3 mL) at the two indicated temperatures. When the MSC-311 dispersion was kept at 46 °C (for 788 min), a MSC-311⇒MSC-322 transformation took place (**a**). When the dispersion was maintained at 15 °C (for 1410 min), MSC-322 slowly transferred back into MSC-311 (**b**). After the dispersion was again returned to 46 °C (for 538 min), the MSC-311⇒MSC-322 transformation was detected once again (**c**). When the dispersion was cooled to 15 °C (for 2490 min), MSC-322 transformed into MSC-311 again, slowly (**d**). The time interval for data collection was 4 min (**a**), 30 min (**b**), 2 min (**c**), and 30 min (**d**). The presence of isosbestic points suggests that the MSC-311⇔MSC-322 transformation is an interconversion. The thermally-induced transformations between MSC-311 and MSC-322 are reversible

Figure 3 presents the absorption spectra collected from a single cyclohexane dispersion but at two temperatures (46 and 15 °C), demonstrating that highly reversible thermally-induced transformations are possible between MSC-311 and MSC-322. The sample cuvette was sealed to ensure that the dispersion was in a closed environment during the whole experimental process. To minimize the presence of the optically transparent IPs, we stored our reaction mixtures until the MSCs ceased to evolve^28^. When a dispersion (with a complete development of MSC-311) was kept at 46 °C (Fig. 3a), the population of MSC-311 persistently declined, while that of MSC-322 increased at the same time. At ~800 min, most of MSC-311 had disappeared, while a significant quantity of MSC-322 had developed. At 15 °C (Fig. 3b), the population of MSC-322 continuously decreased, while that of MSC-311 increased. As a result, MSC-322 appeared to have transformed back into MSC-311. For the cooling of the sample holder (inside the spectrometer) from 46 to 15 °C, it took about one hour (and the dispersion was kept outside of the spectrometer at room temperature (~25 °C)). Therefore, the 788 min spectrum at 46 °C (Fig. 3a) was collected about one hour before the 0 min spectrum at 15 °C (Fig. 3b). This MSC-322⇒MSC-311 reverse transformation at 15 °C took place at a much lower speed than the MSC-311⇒MSC-322 forward transformation at 46 °C. About two days are required for half of the MSC-322 to transform back into MSC-311. For an almost complete conversion from MSC-322 to MSC-311, the dispersion was kept at a slightly higher temperature (room temperature) instead of 15 °C for three additional days.

To study further the reversibility of the transformation, the dispersion discussed above was returned to 46 °C (Fig. 3c). There was a negligible amount of MSC-322 at the start time (0 min). The absorbance of the newly formed MSC-311 (Fig. 3c, 0.5 a.u.) was quite comparable to that of the original MSC-311 (Fig. 3a, 0.5 a.u.). Hence, it appears that the transformation between MSC-311 and MSC-322 was reversible. This reversibility was tested further by placing the dispersion once more at 15 °C (Fig. 3d). For a second time, MSC-322 transformed back slowly into MSC-311. Thus, the dispersion exhibited nearly identical transformations at 46 °C from MSC-311 to MSC-322 (Fig. 3a, c), and at 15 °C from MSC-322 to MSC-311 (Fig. 3b, d).

It is noteworthy that a well-defined isosbestic point was detected in the forward transformation from MSC-311 to MSC-322 at a higher temperature and in the reverse transformation from MSC-322 to MSC-311 at a lower temperature. For reference, isosbestic points were readily detected during molecular isomerization^39^, but have been only reported for one reaction direction for colloidal semiconductor CdSe NCs^47,48^. The detection of the isosbestic point supports our statement that MSC-311 and MSC-322 are one isomeric pair and their thermally-driven reversible solid–solid transformations are direct interconversions without intermediates involved. For the isosbestic points detected (in Fig. 3a, b), Supplementary Fig. 15 shows two expanded views. The isosbestic point located between the two corresponding absorption peaks, and was affected by measurement temperature (such as at ~316 nm/15 °C and ~319 nm/46 °C). There were slight decreases of the optical density at the isosbestic points (and at ~290 nm) from Fig. 3a–c at 46 °C, as well as from Fig. 3b–d at 15 °C. The decrease could be attributed to the probable instability of the MSCs on the experimental time scale with temperature changes. In addition to dispersions, we tested preliminarily the solid-state transformations in their powder forms from MSC-322 to MSC-311 at 15 °C and then from MSC-311 back to MSC-322 at 50 °C. These results were collected from one purified sample and are shown in Supplementary Fig. 16. An isosbestic point was observed also at ~316 nm/15 °C and ~319 nm/50 °C. Importantly, in addition to the synthesis of single-ensemble MSC-311 and MSC-322, the present work has identified them as the first pair of structural isomers of colloidal NCs which exhibit thermally-induced reversible transformations.

### Kinetic study of the reversible transformations between MSC-311 and MSC-322

Kinetic studies have been acknowledged to provide valuable information regarding reaction pathways and mechanisms. Characterization of the kinetics of solid–solid transformations in NCs have been proven to be easier than that for extended solids^1^, and the underlying cause was attributed to the fact that each NC was defect-free by virtue of its size^1^. For a solid–solid transformation in a single NC ~2 nm in size, a single-nucleation event was argued to take place with just one step and without further growth, whereas in extended solids multiple nucleation events were proposed to start at defect sites followed by growth^1^.

Before performing a kinetic study, we examined the relation between MSC concentrations (in cyclohexane or toluene dispersions) and optical absorption. Supplementary Fig. 17 demonstrates that there is clear linear relationship between the concentration of MSC-311 or MSC-322 and the optical absorbance at 311 or 322 nm, respectively. Accordingly, within the concentration range investigated, MSC-311 and MSC-322 appear to be quite stable, with their absorbance following a Beer–Lambert behavior with specific extinction coefficients. Therefore, the concentrations of MSC-311 and MSC-322 can be reasonably quantified by the absorbance $\left( {\varepsilon C_{{\mathrm{MSC}} {\mbox{-}} 311}^t} \right)$ at 311 nm and $\left( {\varepsilon C_{{\mathrm{MSC}} {\mbox{-}} 322}^t} \right)$ at 322 nm, respectively. *ε* is the extinction coefficient of MSCs at their absorption peak positions.

**Fig. 4:**
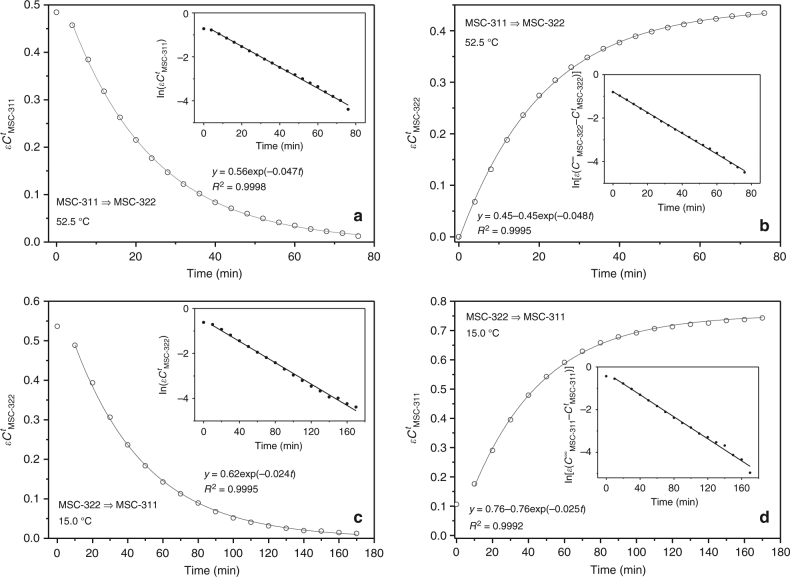
Kinetic study of the transformations at 52.5 and at 15.0 °C. The time-dependent concentrations (open circles) of the reactants (**a**, **c**) and products (**b**, **d**) for the MSC-311 to MSC-322 transformation at 52.5 °C (**a**, **b**) and the MSC-322 to MSC-311 transformation at 15.0 °C (**c**, **d**). Each of the two dispersions was prepared from one reaction mixture (~20 mg from the same approach used for Figs. 1 and 2) in toluene (3 mL). The rate constants obtained are 0.047 min^−1^ (**a**) and 0.024 min^−1^ (**c**), which match well those of 0.048 min^−1^ (**b**) and 0.025 min^−1^ (**d**), respectively

Figure 4 illustrates our kinetic studies of the MSC-311⇒MSC-322 transformation at 52.5 °C (Fig. 4a, b) and the MSC-322⇒MSC-311 transformation at 15.0 °C (Fig. 4c, d), based on the reactants (Fig. 4a, c) and products (Fig. 4b, d). Supplementary Fig. 18 shows the original in situ time-resolved absorption spectra collected from the MSC-311 dispersion in toluene at 52.5 °C and from the MSC-322 dispersion in toluene at 15.0 °C. An isosbestic point has been observed for the MSC-311⇒MSC-322 transformation at 52.5 °C and for the MSC-322⇒MSC-311 transformation at 15.0 °C. The abscissa is the time scale in min, and the ordinate contains the concentration of the reactant (Fig. 4a, c) or product (Fig. 4b, d) at a given elapsed time (*t*). As part of the analysis we performed a mathematical spectral correction for the kinetic study; the details of the mathematical treatment are contained in Supplementary Fig. 19.

During the isomeric transformation, there was a steady decline in the initial reactant concentration, such that it followed closely the behavior of a first-order reaction, as described by Eq. (1).

1 $$C_\lambda ^t = C_\lambda ^0\exp \left( { - k_\lambda t} \right)$$

2 $${\mathrm{ln}}\left( {C_\lambda ^t} \right) = - k_\lambda t + {c}$$

3 $${\mathrm{ln}}\left( {C_\lambda ^0/C_\lambda ^t} \right) = k_\lambda t$$

where $C_\lambda ^t$ is the time (*t*) dependent concentration of MSC-311 at 52.5 °C or of MSC-322 at 15.0 °C, with $C_\lambda ^0$ being the corresponding initial concentration and *k*_*λ*_ the transformation rate constant. For the MSC-311 to MSC-322 transformation at 52.5 °C, the fitted curve of $\varepsilon C_{{\mathrm{MSC}} {\mbox{-}} 311}^t$ vs. *t* according to Eq. (1) (solid curve) indicates a rate constant, *k*_311_, of 0.047 min^–1^ (Fig. 4a). For the MSC-322 to MSC-311 transformation at 15.0 °C, the fitted curve of $\varepsilon C_{{\mathrm{MSC}} {\mbox{-}} 322}^t$ vs. *t* according to Eq. (1) (solid curve) provides a rate constant, *k*_322_, of 0.024 min^–1^ (Fig. 4c). Equation (1) can be converted into Eqs. (2) and (3), with *c* being the constant ${\mathrm{ln}}C_\lambda ^0$. As shown in the insets of Fig. 4, ${\mathrm{ln}}\left( {C_\lambda ^t} \right)$ vs. *t* (solid circles) shows a nearly linear relationship as fitted by Eq. (2) (solid straight lines). This linear relationship could not be verified when the reactant concentration was low, due to the presence of relatively large measurement uncertainty in this range, as illustrated by Supplementary Fig. 20.

To evaluate the reliability of the present kinetic study which is based on in situ time-resolved optical absorption results, we repeated our measurements several times. Supplementary Fig. 21 presents the results of a reproducibility study performed with three repeated experiments on the transformation from MSC-311 to MSC-322 at 46 °C in cyclohexane. The largest variance obtained for the consumption of MSC-311 at each absorbance measurement along the reaction was ∼6% (with an about 95% confidence level). This result indicates that our kinetic study of the MSC-311 to MSC-322 transformation was highly reproducible. In this respect, the concentration of MSC-311 (during the MSC-311 to MSC-322 transformation at 46 °C) also fitted a first-order reaction behavior with a best-fit rate constant, *k*_311_, of 0.017 min^–1^. It is noteworthy that only a few types of chemical reactions exhibit first-order reaction kinetics. They are primarily isomerization and decomposition reactions^39,49^, in addition to the few examples documented for the formation of colloidal semiconductor NCs^1,4,28,41,42^. Of these types of reactions, only isomerization processes^4,39^ can fully account for the “reversibility” demonstrated in Fig. 3.

Figure 4 illustrates that the rate constants of the cluster disappearance (Fig. 4a, c) are similar to those of the cluster formation (Fig. 4b, d). Accordingly, the transformations between MSC-311 and MSC-322 are direct interconversions, with the presence of the isosbestic points indicating mathematically that the rate of MSC-311 concentration decrease should be the same as that of MSC-322 increase (at 52.5 °C) and vice versa (at 15.0 °C). According to the fitting for the MSC-311⇒MSC-322 transformation (Fig. 4a, b), the absorbance was 0.56 for MSC-311, while 0.45 for MSC-322. Accordingly, the initial concentration of MSC-311 (0.56/*ε*_311_) could be considered as similar to the final concentration of MSC-322 (0.45/*ε*_322_), with *ε*_322_/*ε*_311_ = 0.79. Therefore, the overall concentration could be similar. According to the fitting for the MSC-322⇒MSC-311 transformation (Fig. 4c, d), the absorbance was 0.62 for MSC-322, while 0.76 for MSC-311. Accordingly, the initial concentration of MSC-322 (0.62/*ε*_322_) could be considered as close to the final concentration of MSC-311 (0.76/*ε*_311_). Thus, the kinetic study of the thermally-induced reversible MSC-311⇔MSC-322 transformations supports that they are a pair of isomers. For solid–solid structural transformations in nano-scale functional materials (within the size of nuclei)^1,20,28^, such single-nucleation events with first-order reaction kinetics may generally occur.

### Barrier energy of the MSC-311 to MSC-322 transformation

**Fig. 5:**
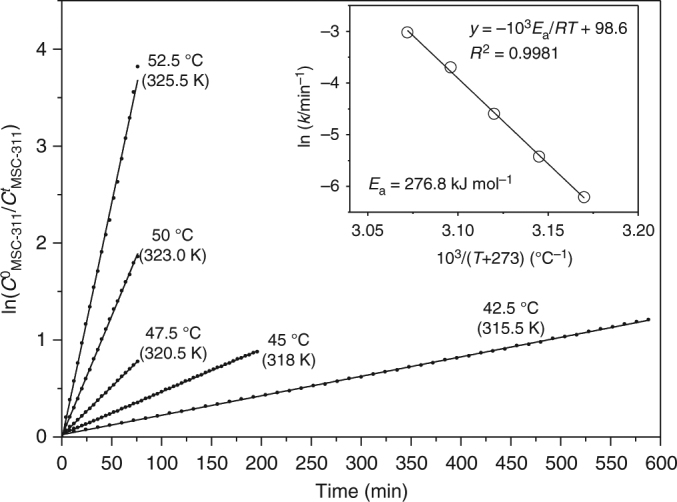
First-order reaction kinetics fitting for the MSC-311⇒MSC-322 transformations. Five identical MSC-311 dispersions in toluene (3 mL) were made from a single MSC-311 sample (~20 mg). The five dispersions were placed at the five temperatures (*T*) as indicated from 42.5 to 52.5 °C. The time-dependent quantity ${\mathrm{ln}}\left[ {C_{{\mathrm{MSC}} {\mbox{-}} 311}^0/C_{{\mathrm{MSC}} {\mbox{-}} 311}^t} \right]$ (solid circles) fits well with first-order reaction Eq. (3) (solid lines). $C_{{\mathrm{MSC}} {\mbox{-}} 311}^0$ represents the starting concentration of MSC-311 and $C_{{\mathrm{MSC}} {\mbox{-}} 311}^t$ is the time-dependent concentration of MSC-311 during the transformation. The slopes of the fitted lines correspond to the rate constants *k* (min^−1^). The inset shows the Arrhenius plot, with a *y*-axis of ln(*k*) and an *x*-axis of 1/*T*. The slope of the fitted line is *E*_a_/*R*, where *E*_a_ is the barrier energy and *R* the ideal gas constant

For the MSC-311 to MSC-322 transformation, the rate constant was observed to be quite sensitive to temperature. To calculate the activation energy of the transformation, we applied the Arrhenius equation to the unimolecular reaction associated with the transformation. Figure 5 shows the time dependence of the quantity of ${\mathrm{ln}}\left[ {C_{{\mathrm{MSC}} {\mbox{-}} 311}^0/C_{{\mathrm{MSC}} {\mbox{-}} 311}^t} \right]$ (solid circles) for the transformations in toluene at five temperatures ranging from 42.5 to 52.5 °C. It is apparent that the solid lines, generated from the first-order reaction kinetic fits obtained using Eq. (3), are in good agreement with the experimental data (solid circles). The Arrhenius plot, ln *k* = −*E*_a_/*RT*, is shown in the inset, with *R* the gas constant and *T* the temperature. The Arrhenius plot indicated a barrier energy, *E*_a_, of 276.8 kJ mol^–1^. Here, the original absorption spectra used can be found in Supplementary Fig. 22.

We also explored the barrier energy for the MSC-311 to MSC-322 transformation in cyclohexane, as shown by Supplementary Fig. 23 (with the original optical absorption spectra collected from four identical MSC-311 dispersions, each at a different temperature). From the rate constants obtained, the Arrhenius plot gave the barrier energy, *E*_a_, of 269.3 kJ mol^–1^, which is identical within uncertainty to that obtained from the toluene dispersions. For the MSC-311 to MSC-322 transformation in toluene and cyclohexane, the rate constants measured were different at the same temperatures (such as 47.5 or 50.0 °C and 52.5 °C). The transformation occurred generally faster in cyclohexane than in toluene at the same temperature^1^. However, the energy barrier calculated was quite similar for the two solvents used, which suggests that the breaking of the Cd–S bond could be the probable rate-determining step^40^.

## Discussion

Very recently, we developed and reported an effective two-step approach to colloidal compound semiconductor MSCs. This approach enables production of the MSCs in a single-ensemble form without the simultaneous production of other-size NCs^27,28^. The key point of the approach is to stop a reaction in its induction phase, which is prior to nucleation and growth of RQDs. We proposed there was a general pathway, the so-called Yu pathway, which leads to the formation of both MSCs and RQDs via a repeated process of proton-mediated ligand exchange^27,28,38^. When cation (M) and chalcogen (E) precursors are mixed, the noncovalent interactions between the two precursors result in self-assembled clusters of ~1 nm in size. For these micellar-like aggregates, covalent M–E bonds are not present. As the reaction progresses, the proton-mediated ligand exchange results in the formation of covalent M–E bonds inside the micellar-like aggregates, and the resulting IPs can directly transform to MSCs through a structural transformation process. The IP is optically transparent without an ordered structure and has a similar size to that of the micellar-like aggregates. We therefore proposed that the formation of CdS MSC-311 experiences a two-step process and the IP to MSC transformation follows first-order unimolecular reaction kinetics^28^.

**Fig. 6:**
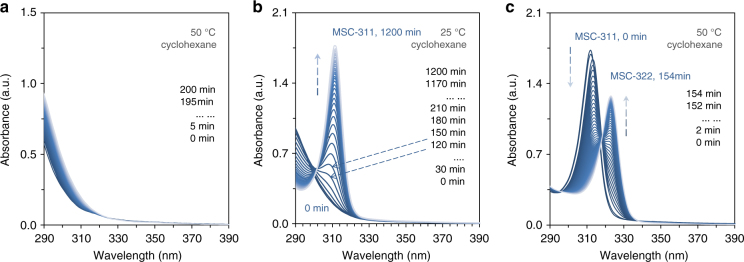
In situ time-resolved absorption spectra collected from one cyclohexane dispersion. When the sample (~31 mg prepared similar as that for Fig. 1) was dispersed in cyclohexane (3 mL) at 50 °C (**a**), there was a limited change in absorbance observed at 322 nm for up to 200 min (from dark blue to gray). When this dispersion was cooled to 25 °C, MSC-311 started to develop around 2 h (**b**). When this dispersion (after 20 h at 25 °C) was then placed at 50 °C, a MSC-311⇒MSC-322 transformation readily took place (**c**). Therefore, the evolution of MSC-322 via the solid–solid phase transformation from MSC-311 in dispersion at 50 °C (**c**) appears to be much more efficient than that the liquid–solid phase transformation from the IP in dispersion at 50 °C (**a**)

Clearly, the development of MSC-311 from the optically transparent IP takes place advantageously at a relatively low temperature, when compared to the evolution of MSC-322 from the same IP. The two-step evolution for MSC-311 from an IP dispersion at room temperature 25 °C occurs more readily than a similar process occurs for MSC-322 at 50 °C. Fig. 6a shows the in situ absorption spectra collected from one IP dispersion in cyclohexane at 50 °C. Note that 50 °C is a temperature that favors the formation of MSC-322 directly from a reaction mixture that has been pre-heated at 180 °C for example. However, the development of MSC-322 directly from the IP in the dispersion was apparently very slow at 50 °C, as there was little increase of the absorbance at 322 nm observed for times up to 200 min. When the dispersion was cooled to 25 °C (Fig. 6b), MSC-311 started to develop from the IP within ∼60 min; the continuous absorbance increase at 311 nm was accompanied by a parallel absorbance decrease at ~290 nm. When the dispersion with well-developed MSC-311 (for a period of 1200 min at 25 °C) was subsequently heated to 50 °C (Fig. 6c), almost all MSC-311 converted to MSC-322 within approximately two and a half hours.

From the observations discussed above, there would appear to be two pathways to formulate MSC-322 dispersions at 50 °C. One is IP⇒MSC-322. The other is IP⇒MSC-311⇒MSC-322, which involves a MSC structural transformation. The results in Fig. 6 indicate that the former pathway (Fig. 6a) is evidently much less efficient than the latter one (Fig. 6b, c); high dispersion temperatures seem to be less effective than low dispersion temperatures for the IP to MSC transformation. The relatively high temperature of 50 °C does not favor the formation of MSC-322 directly from the IP, while the relatively low temperature of 25 °C facilitates the formation of MSC-311. Afterwards, MSC-311 transfers readily to MSC-322 by structural isomerization at 50 °C. Such an indirect MSC-311⇒MSC-322 pathway proves to be more efficient than the direct IP⇒MSC-322 pathway. Structural isomerism could be a beneficial general route to produce NCs^12^.

As indicated by mass characterization with MALDI-TOF MS and TGA, together with structural characterization with PDF analysis of X-ray total scattering, XAFS, SAXS, WAXS, and XRD, MSC-311 and MSC-322 appear to have the same cluster mass and composition, and a very much similar local structure but slightly different surface structures. The surface variance could be due to shape difference, similar to that reported for TiO_2_^50^. Due to high surface-to-volume ratios of small-size clusters, the surface structural difference could play an important role, influencing properties, such as bandgap and thermostability. MSC-311 and MSC-322 are thus the first pair of isomers identified for semiconductor NCs. It seems reasonable that the cluster surface plays a key role for the cluster transformation detected. Furthermore, their thermally-induced reversible transformations are direct interconversions without intermediates being involved, as supported by the fact that the forward and reverse transformation has an isosbestic point. For the II–VI semiconductor bulk and RQDs, thermally-induced phase transformations from zinc-blende to wurtzite structures have been documented. The two structures, however, have nearly identical bandgap energies, and optical absorption spectroscopy has not been shown to be a practical tool to meticulously monitor the transformation kinetics of the bulk materials and RQDs^51–55^. On the other hand, for the two types of MSCs discussed in the present work, there is a significant difference in bandgap energies. Subsequently, we were able to effectively perform in-depth kinetic studies for the cluster transformations using in situ optical absorption spectroscopy. We have demonstrated that the thermally-induced structural isomerism between MSC-311 and MSC-322 is reversible and follows closely first-order unimolecular reaction kinetics. Such kinetic behavior has been reported for the pressure-induced solid–solid transformation in small-size semiconductor NCs and for the CdS IP-311 to MSC-311 structural transformation under ambient conditions^1,4,28^. We anticipate that such kinetics could also occur for the isomerism of other nanoparticles (NPs) including Au clusters^13^.

For the MSC-311 to MSC-322 transformation, the energy barrier *E*_a_ obtained was ~277 kJ mol^–1^ in toluene and ~269 kJ mol^–1^ in cyclohexane. The value of an energy barrier is usually related to the rate-determining step of a reaction. It is noteworthy that the dissociation energy of a Cd–S bond is ~200 kJ mol^–1^ in the bulk CdS^40^. For this reason, the energy barrier that we have obtained suggests that the rate-determining step of the transformation possibly involves the breaking of Cd–S bonds. The breaking of Cd–Se bonds was reported to take place under ambient conditions in the cation exchange reaction from CdSe to Cu_2_Se^56,57^. Therefore, the MSC-311 to MSC-322 transformation in the present study could be occurring through the breaking of Cd–S bonds. The formation of MSC-311 could also be induced by alcohols from IP-311^28^ or MSC-322^31,58^. Such formation and transformation has been suggested to be surface-related by accumulated experimental evidence^1,26–28,31,37,41,48,58^.

In conclusion, we have presented compelling evidence regarding the identification of a pair of isomers of colloidal compound semiconductor NCs, CdS MSC-311, and MSC-322. The two types of MSCs have the same cluster mass, a similar local structure, but slightly different surface structures (due to shape)^50^. Their thermally-induced solid–solid transformation is reversible and repeatable, following first-order unimolecular reaction kinetics. The present study suggests that it is practical to fine tune the properties of nano-scale materials through surface structural changes^59^. Such changes may, for example, allow the nano-scale materials to act as bits in information storage or other applications. An in-depth understanding of the structural isomerism would be essential to design and engineer advanced miniaturized devices, whose switching kinetics could be accelerated or delayed as per the requirement of individual applications. We anticipate that the present work will inspire and motivate more studies (including modeling) on structural transformations of colloidal NCs^47,60^.

## Methods

### Chemicals

All chemicals are commercially available and used as received without purification (except stated otherwise). Cadmium oxide (CdO, 99.99%), oleic acid (OA, 90%), ODE (90%), and bis(trimethylsilyl)sulfide ((TMS)_2_S) were purchased from Sigma-Aldrich. Cadmium acetate dihydride (Cd(OAc)_2_·2H_2_O, 99.999%) and the bulk cadmium sulfide (CdS, 98%) were from Alfa Aesar. Powder sulfur (S, 99.5%) and isopropanol (i-PrOH, 99.7%) were received from Chengdu Ke Long Chemical. Phenylacetic acid (PhCH_2_COOH, 98%) was from Sinopharm Chemical Reagent. DCTB (98.0%) and CHCA were obtained from Tokyo Chemical Industry. Toluene (99.5%) and cyclohexane (CH, 99.5%) were from Chengdu Ke Long Chemical; dried solvents were used for the kinetic study (with ~20 g of Na_2_SO_4_ for 1000 mL of solvents).

### Synthesis of CdS MSC-311 and MSC-322 passivated by oleate ligands

The preparation of our Cd precursor, Cd(OA)_2_ stock solution, can be found elsewhere but with modification^61^. A mixture of CdO (6.00 mmol) and OA (13.20 mmol) in 5 g of ODE was placed in a 50 mL three-necked reaction flask. CdO was dissolved completely at 240 °C and then cooled to 120 °C in a nitrogen (N_2_) atmosphere. The as synthesized Cd(OA)_2_ stock solution was vacuumed for 60 min to remove water. The reaction started with a batch consisting of Cd(OA)_2_ (0.60 mmol from the stock solution), elemental S (0.15 mmol), and ODE (4 g). Based on the newly developed two-step approach, in the first step, the mixture was heated up to about 180 °C in N_2_ for 20 min; the mixture was cooled and stored at –20 °C or 4 °C for the formation of MSC-311 and at 30, 60, or 80 °C for the formation of MSC-322 in the second step. In the kinetic study, the reaction mixture from the first-step was dispersed in 3 mL of toluene or cyclohexane at room temperature in the second step. MSC-322 could be obtained by indirect method also, from heating a MSC-311 dispersion at 50 °C; inversely, MSC-311 can also be obtained by keeping a MSC-322 dispersion at a temperature lower than 25 °C. For purification, ∼160 mg of the sample (stored sample) was added to a mixed solvent consisting of ~3 mL of isopropanol and 1 mL of toluene. After centrifugation, the supernatant was removed and the precipitate was dried under vacuum to remove residual isopropanol quickly to avoid the presence of RQDs, which sometimes did present.

### Ultraviolet absorption spectroscopy

Absorption spectra were collected with Beijing Purkinje General TU1901 apparatus or a TECHCOMP UV 2310 II ultraviolet–visible (UV–vis) spectrometer, with a 1-nm data collection interval.

### Matrix-assisted laser desorption/ionization time of flight mass spectrometry

The Bruker Autoflex III MALDI-TOF mass spectrometer was used to collect the mass spectra of the sample. The measurements were done in the Key Lab of Analytical Chemistry for Life Science in the Institute of the Chemistry Chinese Academy of Sciences and Key State Laboratory and Supramolecular Structures and Materials of Jilin University. DCTB or CHCA was used as a matrix and 10 mg stock solution was dispersed in 0.5 mL of toluene. 10 μL of as-synthesized CdS reaction solution was dispersed in 500 μL of toluene. To enhance the signal, the matrix solution and CdS sample solution were mixed in a 1:1 volume ratio. The mixture was then smeared onto a MALDI plate and loaded into the MALDI chamber for characterization with the detector gains (linear detector, positive mode) set at 7.2× or 10.0×.

### X-ray total scattering experiments

Total scattering diffraction experiments were performed using a PANalytical Empyrean instrument in a transmission mode with Ag Kα (wavelength 0.559 Å) radiation, a Rh Kβ filter and a GaliPIX^3D^ detector. The samples were loaded in 1 mm glass capillaries. The diffraction data were then collected in the 2 theta range from 2° to 140° in 12 h. The raw powder diffraction data were Fourier transformed to PDF using X’Pert HighScore^62^ with a *Q*_max_ of 15 Å^–1^. The real space data were modeled using PDFgui^63^. The *Q*_damp_ and *Q*_broad_ were calibrated by refining the structure of a silicon sample. Details of the PDF analysis can be found in Supplementary Method.

### Data availability

The authors declare that all relevant data supporting the findings of this study are available from the authors on request.

## Electronic supplementary material

Supplementary Information
