# Supplementary material for: Thermally-induced reversible structural isomerization in colloidal semiconductor CdS magic-size clusters
Source: Nat Commun. 2018 Jun 27;9:2499. doi: 10.1038/s41467-018-04842-0 (PMC6021431; doi:10.1038/s41467-018-04842-0)
Supplement: Supplementary file 1 — Supplementary Information [file 41467_2018_4842_MOESM1_ESM.pdf]

Thermally-induced reversible structural isomerization in colloidal semiconductor CdS magic-size clusters

Kui Yu* *et.al.*

Supplementary Methods. Atomic pair distribution function (PDF) analysis.

The atomic pair distribution function (PDF), $G(\mathrm{r})$, is the Fourier transform of a total scattering structure function $S(Q)$, where

$$G(\mathrm{r}) = 4\pi r[\rho(r) - \rho_0] = \frac{2}{\pi}\int_0^\infty Q[S(Q) - 1]\sin(Qr)dQ. \quad (1)$$

G(*r*) is related to the probability of finding an atom at a given distance from another one[1,2]. $S(Q)$ is obtained from the corrected and normalized powder diffraction intensity. $\rho(r)$ is the atomic pair density function, ρ_0 is the atomic average number density, and *Q* is the magnitude of momentum transfer on scattering. PDF analysis has been applied to explore the structure of nanomaterials[3-5]. For Supplementary Fig. 7, the real space diffraction data, $G(\mathrm{r})$, were fitted with PDFgui[6], in order to estimate the range of structural coherence of CdS bulk and RQDs. RQDs were assumed to be spherical in shape with the diameter of *D*. Accordingly, the attenuated bulk pair distribution function can be written as

$$G(r) = G(r)_{\mathrm{bulk}}\, \gamma_0(r),$$

$$\gamma_0(r) = \left[1 - \frac{3r}{2\mathrm{D}} + \frac{1}{2}\left(\frac{r}{D}\right)^3\right] H(D - r) \quad (2)$$

where H is a Heaviside step function[7,8].

Supplementary Table 1. Summary of the solid-solid structural transformation of semiconductors reported and of the CdS MSCs in the present study.

S-S Trans.[a]	Materials, Domain size	Optical Absorption	Induced conditions	Kinetics	Reversible situation	Ref
Wz[b] ⇔ Rs[c] bulk	CdSe/CdS, > 60 nm	broad ⇔ none	Pressure 3.5 GPa	Multi-site nucleation, followed by growth	Size decreased, Partially recover (with cubic)	9,10
Wz ⇔ Rs Medium-size NCs	CdSe/CdS, > 4.3 nm	broad ⇔ none	Pressure 3.5–10 GPa	Single-site nucleation, followed by growth	No size change, Partially recover	11
Wz ⇔ Rs Small-size NCs	CdSe/CdS, ~ 2.0 nm	broad ⇔ none	Pressure 3.5–10 GPa	Single-site nucleation, uni-molecular first order	No size change, Partially recover	11,12
Wz ⇔ Rs $Cd_{32}S_{50}$	CdS, 1.5 nm	broad ⇔ none	Pressure 3.5–10 GPa	Single-site nucleation, uni-molecular first order	No size change, Partially recover	13
MSC–311 ⇔ MSC–322	CdS, ~ 2.0 nm	sharp ⇔ sharp	Thermal 15–60 °C	Single-site nucleation, uni-molecular first order	No mass change, Totally recover	Present study

[a] S-S Trans: solid-solid transformation. [b] Wz: wurzite structure. [c] Rs: Rock-salt structure.

Supplementary Table 1 illustrates that, for a bulk material (grain size > 80 nm CdSe), it was suggested that the transformation from wurtzite to rock-salt structures started at defect sites with multiple nucleation events. Due to the presence of defects, the barrier height in that case was lower than that of a NC.[9-11] For a medium-size NC (such as ~4.3 nm CdSe), its wurtzite to rock-salt transformation featured a single nucleation event followed by growth within its finite size.[9-11] For a small-size NC (such as ~3.4 nm CdSe), its wurtzite to rock-salt transformation proceeded with a single nucleation event but without further growth. The coherent deformation of the entire nanocrystal was considered to take place in a single step[11,12]. This phenomenon could be understood by the size of critical nuclei which was larger than NCs, which consisted of only 10^2 to 10^3 atoms (as suggested for the solid-solid phase transitions in the bulk).[11] Here, the transformation followed first-order unimolecular reaction kinetics[11,12]. For all the sizes investigated, the rock-salt phase transformed back to the wurtzite phase when the pressure was decreased, but with the coexistence of a zinc-blende phase and little change in the grain sizes of the NCs. For the bulk materials, the grain sizes decreased[9-12].

For a very small cluster of the size of ~1.5 nm, such as a magic-size cluster (MSC) $Cd_{32}S_{14}(SPh)_{36} \bullet DMF4$ (DMF = N,N-dimethyformamide), its pressure-induced transformation from zinc-blende to rock-salt structures proceeded via nucleation with a small barrier which is similar to those associated with molecular isomerization.[11] This MSC exhibits a sharp absorption peak at 358 nm (at room temperature in tetrahydrofuran),[13] and is denoted by MSC-358. The pressure-induced phase transformation of MSC-358 was demonstrated to be reversible, with some sensitivity to dispersion solvents used. It was suggested that bond breaking and/or rearrangement between surface atoms and capping ligands dominated the kinetics[11].

Supplementary Table 2. Composition estimation for our $PhCH_2COO$-passivated MSCs, based on MALDI-TOF MS, TGA and ICP-OES[a].

Samples	Ligand (%) TGA	Cd/S ratio ICP-OES	Cd (%)	S (%)
MSC-311 (mass)	45.9	8.4	48.3	5.8
MSC-311 (molar)	35.8	2.4	45.3	18.5
MSC-322 (mass)	45.2	8.6	48.9	5.8
MSC-322 (molar)	34.7	2.5	46.3	18.9
CdS Bulk (mass)		4.6	74.8	16.4
CdS Bulk (molar)		1.3	56.5	43.5

[a]Inductively Coupled Plasma Optical Emission Spectroscopy (ICP-OES) was measured with SPECTRO ARCOS FHS12 instrument in Sichuan Univ. Samples were digested in concentrate nitric acid, and then diluted with 2% nitric acid for analysis (~50 mg in 50 mL). From the value of 1.30 obtained for commercial bulk CdS (with 98% purity) which was used as a “control/standard” sample, it is reasonable that ICP-OES has its own limitation for the characterization of the Cd to S atomic ratio of CdS samples.

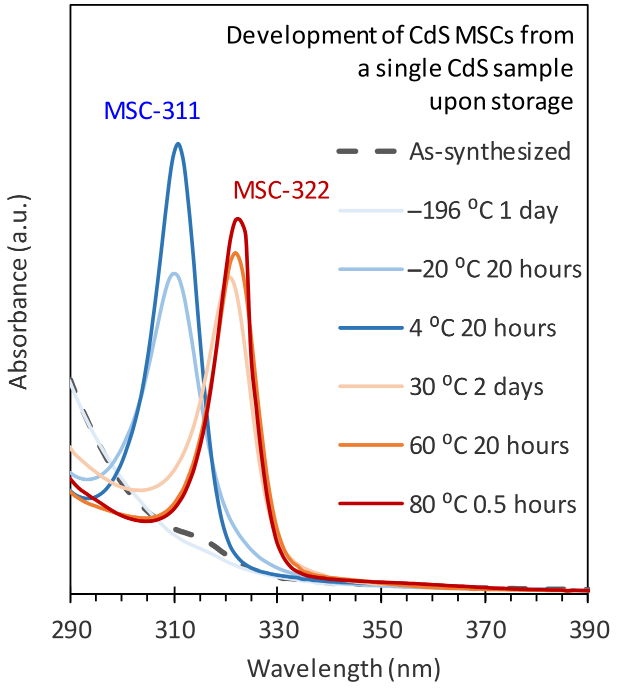

Supplementary Figure 1. Optical absorption spectra collected from a single reaction mixture (~20 mg) which was preheated at 180 °C and then incubated at 6 different temperatures with several well-defined periods of time, as indicated. The solvent used was cyclohexane (3 mL). The preparation of the reaction mixture was similar to that described in Fig. 1. The as-synthesized sample seemed to be optically transparent (dashed gray trace). As no change in the absorption spectrum occurred at –196 °C over a relative long storage period (24 hours), such a liquid nitrogen environment was used to store samples for future usage, such as for the samples used for Figs. 3 and 6. When a sample was stored at –20 °C or 4 °C for 20 hours, CdS MSC-311 developed (light and dark blue traces, respectively), whereas when stored at 30 °C for 48 hours, 60 °C for 20 hours and 80 °C for 0.5 hour, CdS MSC-322 developed (light, medium and dark orange traces, respectively).

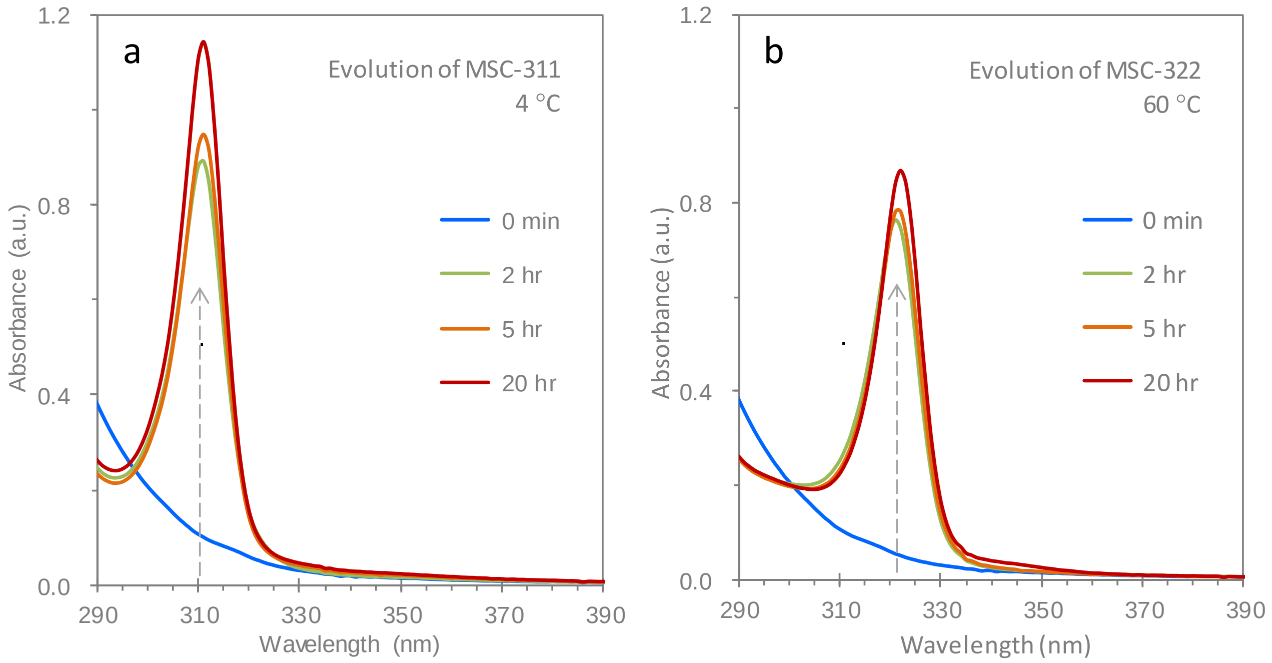

Supplementary Figure 2. Temporal evolution monitored by optical absorption spectroscopy of CdS MSC-311 (a) and MSC-322 (b) from a single reaction mixture stored at 4 °C (a) and at 60 °C (b). The elapsed periods are indicated. The solvent used was cyclohexane. The preparation of the reaction mixture was similar to that described in Fig. 1 and Supplementary Fig. 1. The consumption of the IP, in the optically-transparent as-synthesized sample (blue traces), leading to the development of the MSCs, was relatively fast in the first two hours.

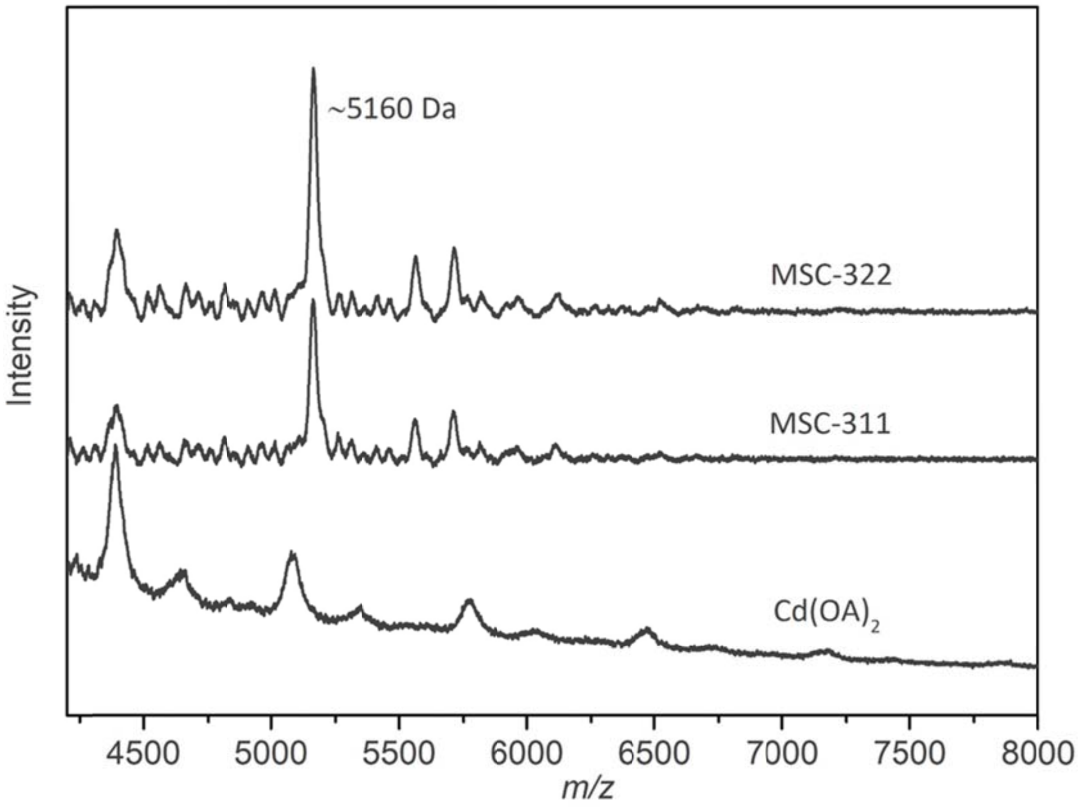

Supplementary Figure 3. MALDI-TOF mass spectra collected from $Cd(OA)_2$, MSC-311 and MSC-322 samples. This experiment was similar to that for which the results are shown in Fig. 2a, except that a different matrix, CHCA (α-Cyano-4-hydroxycinnamic Acid), was used. Species with a *m/z* value of approximately 5160 Da (or 5.16 kDa) were detected for both MSC-311 and MSC-322. Peaks at regular *m/z* intervals were detected from $Cd(OA)_2$. We have to point out that, if we follow the literature[14] with kDa as the unit instead of Da for mass, then, the mass with more significant numbers obtained was 5.159741 kDa for MSC-311 and 5.158417 kDa for MSC-322, with DCTB as the matrix (Fig. 2a). With CHCA as the matrix, the mass obtained was 5.162503 kDa for MSC-311 and 5.163844 kDa for MSC-322. The mass measurement would suggest different compositions for a core-cage structure with a stoichiometric composition[15] and for a tetrahedral structure with a non-stoichiometric composition[16].

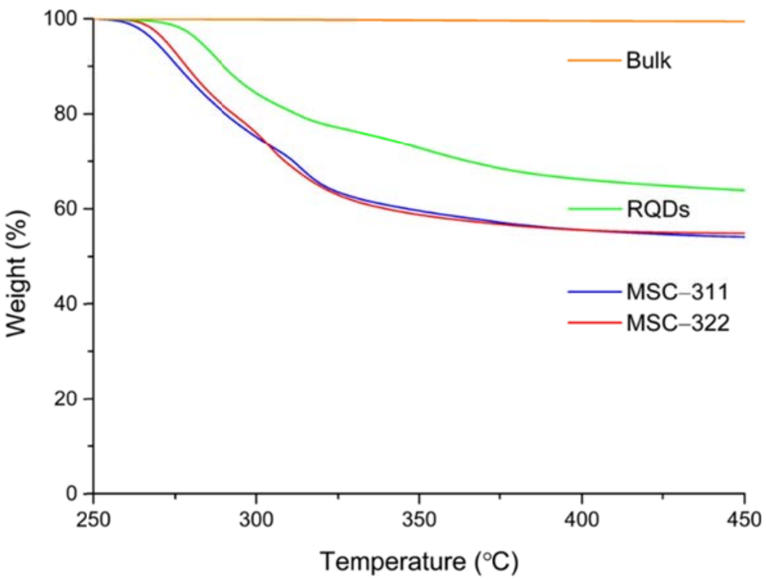

Supplementary Figure 4. Thermogravimetric Analysis (TGA) of MSC-311 (blue trace), MSC-322 (red trace), CdS RQDs (green trace) and CdS bulk (yellow trace). All samples (10-20 mg) were dried under vacuum to remove solvent first. The TGA measurements were performed by NETZSCH STA 449C Instruments under a nitrogen atmosphere, with the temperature increase of 15 °C/min from 30 °C to 450 °C. The TGA result suggests that the weight ratio of organic ligands to inorganic ratios of 45.9 to 54.1 for MSC-311 and 45.2 to 54.8 for MSC-322. Based on such similar values in combine with the similar mass result and PDF study, it is reasonable that the inorganic cores of the two clusters have the same local structure with the mass. The two clusters are a pair of isomers.

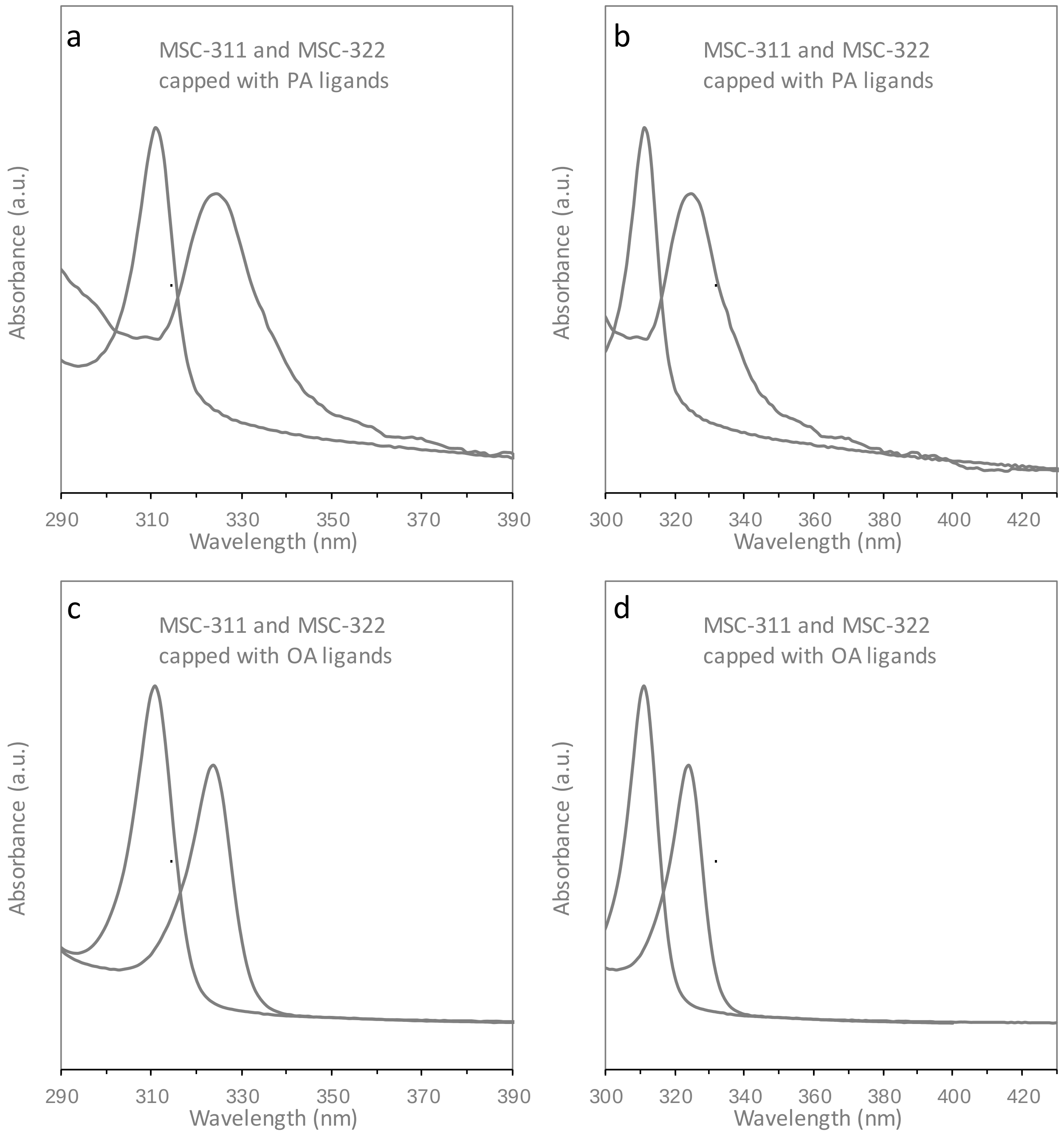

Supplementary Figure 5. UV-vis spectra of our MSC-311 and MSC-322 passivated by phenyl acetate (PA) ligands and (purified in toluene after 5 min of sonication) (a, b) and by oleate (OA) ligands (unpurified in toluene) (c, d). The wavelength windows were 290-390 nm (a, c) and 300-430 nm (b, d). The PA passivated MSCs exhibit more spectrum broadening and scattering at longer wavelengths; the cause may be related to relatively low solubility due to

relatively more ready π-π stacking of the surface ligands. The possible stacking is in agreement with our small-angle X-ray scattering (SAXS) shown in Supplementary Fig. 11. The purified PA passivated samples were used for the structural characterization (presented by Fig. 2b and Supplementary Figs. 6-13). The following procedure was followed to synthesize the MSCs passivated by phenyl acetate ligands. (1) A mixture of $Cd(OAc)_2$ (0.60 mmol) and $PhCH_2COOH$ (2.16 mmol) was placed in a 50 mL three-necked reaction flask. (2) The reaction mixture was then heated to 90 °C, producing a colorless liquid, to which 1 mL of toluene was added to prepare the Cd precursor ($Cd(OOCCH_2Ph)_2$). (3) The Cd precursor was maintained at 60 °C (for MSC-311) or 90 °C (for MSC-322), and a mixture of $(TMS)_2S$ (0.30 mmol), and toluene (1 mL) was injected under a N_2 atmosphere. (4) The reaction was allowed to proceed at the injection temperature for ~5 minutes. (5) The mixture was cooled down in an ice-water batch to produce MSCs. When the injection temperature was 110 °C, RQDs formed. For the purification of the CdS MSCs, the as-synthesized reaction solution (~5 g) was centrifuged directly, whereupon the MSCs precipitated in a white powder at the bottom of the tube. The precipitate was then washed twice by ~500 μL of toluene to remove the unreacted precursors. The precipitate was quickly dried under vacuum with the residual toluene removed. For the purification of the CdS RQDs, approximately 5 g as-synthesized sample was added to 15 mL of methanol for centrifuging. Again, after centrifugation, the supernatant was removed and the precipitate was dried with the residual solvent removed quickly under vacuum.

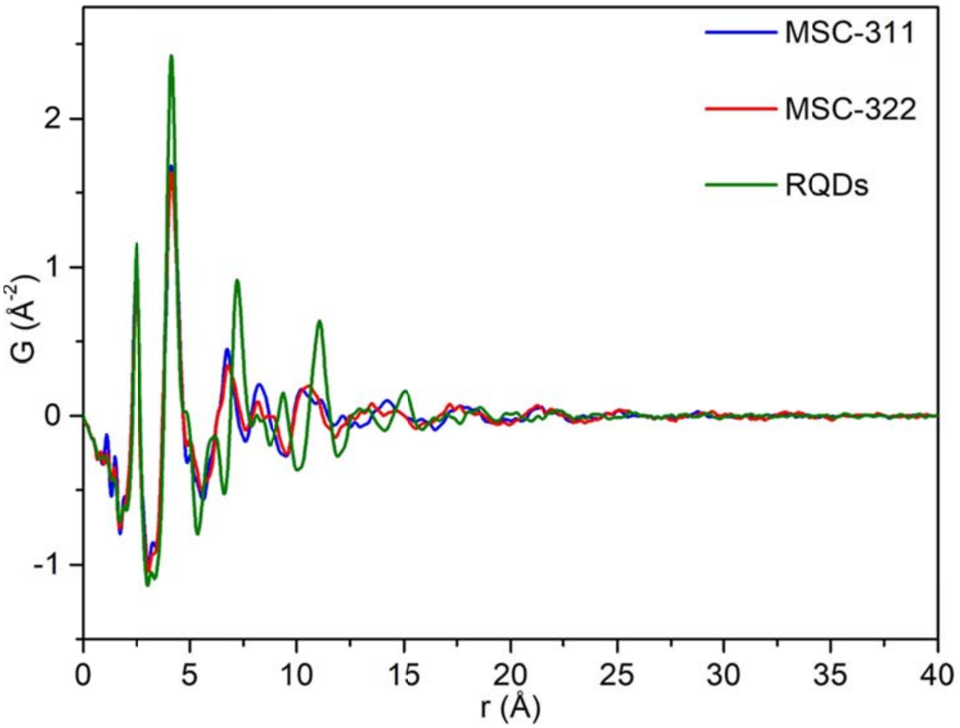

Supplementary Figure 6. PDF results for CdS MSC-311 (blue trace), MSC-322 (red trace) and RQDs (green trace, passivated by phenyl acetate ligands with bandgap absorption at 363 nm). The PDFs are dominated by Cd and S correlations and the ligands have no noticeable presence within the PDFs. The three samples exhibit similar peak positions for the first two peaks in the short-r region (< 5 Å), which is not unexpected because the first two peaks represent the nearest Cd-S, Cd-Cd and S-S distances. In the range of 5 to 10 Å, the three samples exhibit different PDF patterns regarding peak positions and intensities, implying structural differences. The sharp correlations are indicative of the diameter of 10 – 25 Å for the three NCs. The broad long-range particle-to-particle correlations (as seen by the relatively broad peaks with reducing intensities) in the long-r region survives up to ~30 Å. The PDF analysis suggests that the two clusters have a similar size (based on the gradual decline in amplitude of the ripples in the PDFs at the long-r distance), but different surface structures, according to that reported for TiO_2[17]. The PDF results are not practical to calculate the size of small nanocrystals (NCs), due to the lack of models. But, we tried for the RQDs, using PDFgui program[6] under the assumption of a spherical shape with a two-phase

model (as shown in Supplementary Fig. 7). The value of 2.2 nm was obtained. Accordingly, the two cluster sizes should be smaller than 2.2 nm.

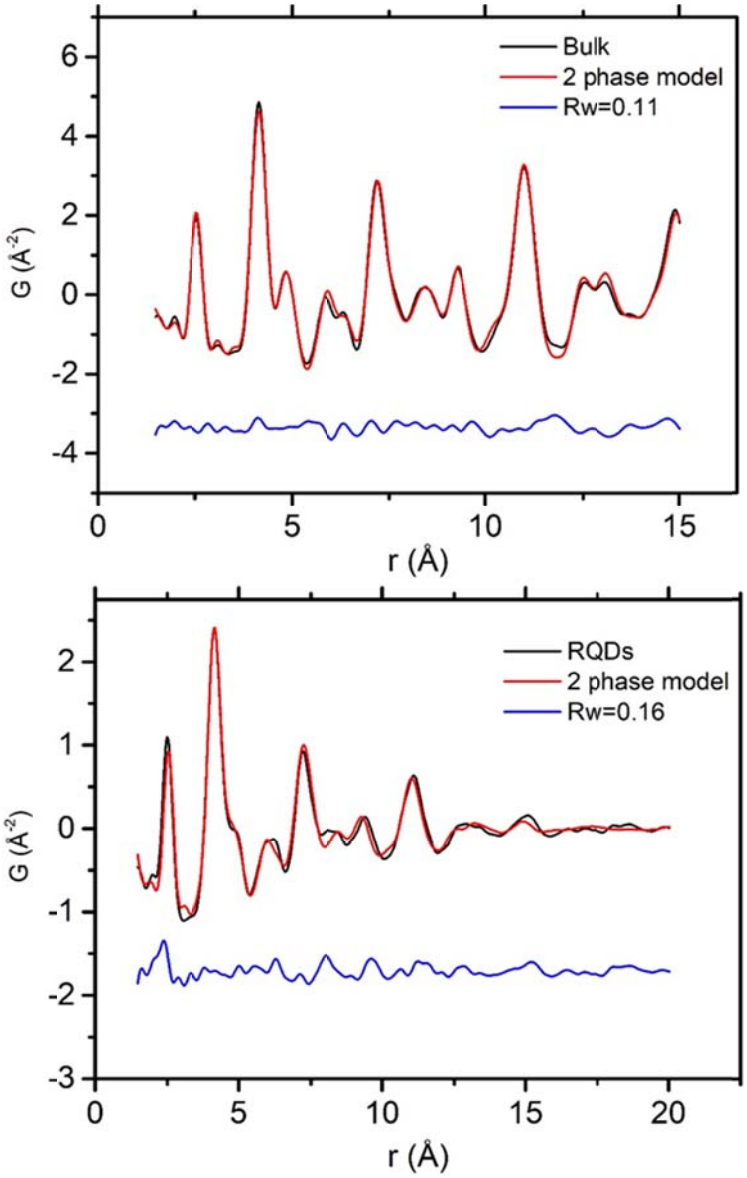

Supplementary Figure 7. PDF analysis of CdS bulk (top, black trace) and RQDs (bottom, black trace), and their simulated PDF (top and bottom, red traces). For the simulations (red traces), we used a two-phase model (with zinc-blende $F\bar{4}3m$ and wurtzite $P6_3mc$ phases) together with a shape function that represents the finite range of interatomic distances within a cluster. The two fits (red traces) are in good agreement with the data (black traces), as suggested by the two low residuals (blue traces) and goodness of fit values ($R_w < 0.16$).

We interpret the use of the two-phase model as best representing the effects of stacking faults within the RQDs.

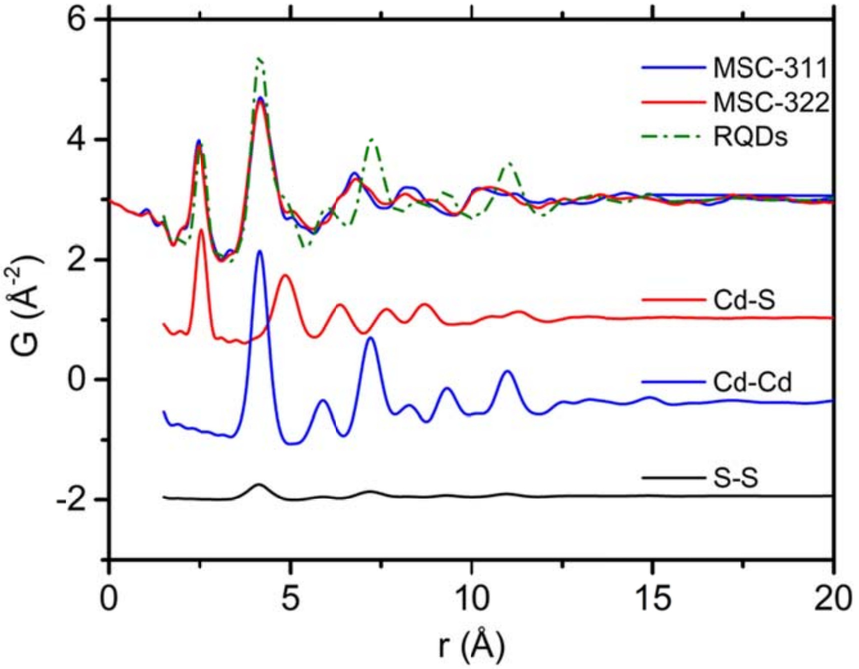

Supplementary Figure 8. Partial pair distribution functions (PDFs) of S-S (black trace), Cd-Cd (blue trace) and Cd-S (red trace) correlation obtained, from the PDF simulation of the RQDs (shown in Supplementary Fig. 7). For comparison, the PDF data of CdS MSC-311 (dark blue trace), MSC-322 (dark red trace) and RQDs (dashed green trace) are also presented. As indicated, the first peaks (at ~2.46 Å) of the three samples originated from the nearest-neighbour Cd-S correlation, while the second peaks (at ~4.15 Å) are from nearest-neighbour Cd-Cd and S-S correlations.

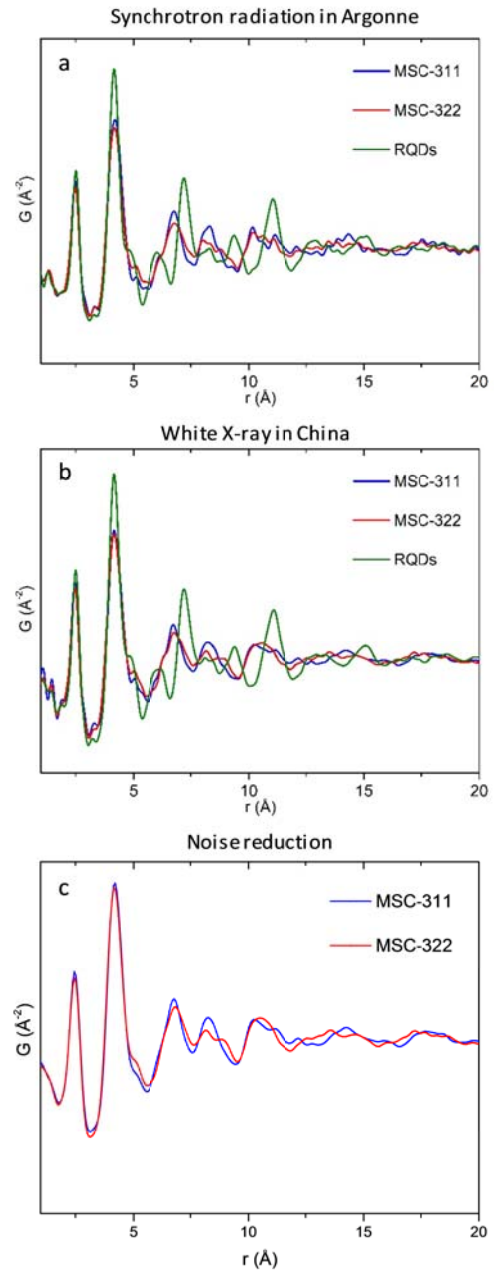

Supplementary Figure 9. (a) PDF analyses based on the total scattering experiments performed in Argonne National Laboratory (beam line 11-ID-C, with high-energy X-rays of 105.7 keV photon energy) for the same two clusters as indicated. The GSAS-II program[18] was used for the PDF analysis. (b) PDF analysis based on the in-house white X-ray. (c) Pair distribution functions G (G(r) is referred to as D(r) in some circumstance) for the same total scattering in (b), obtained by sine transform of the Qi(Q) functions. We used Q_{max} = 15 $Å^{-1}$. The data were multiplied by the Lorch modification function $M(Q) = \sin(Q/Q_{max})/(Q/Q_{max})$ prior to transform to reduce the size of the termination ripples in the Fourier transform and to reduce the effect of noise in the data at higher Q. We also interpolated the low Q data down to Q = 0. In conclusion, it is clear that the sharp peaks at 7.2, 9.2 and 11.1 Å for the RQDs withdraw evidently for the two clusters MSCs. These three sharp peaks represent the Cd–Cd distances beyond the first-nearest-neighboring CdS_4 tetrahedral. Accordingly, the MSC-311 and MSC-322 have a similar CdS_4 tetrahedron local structure, but with different intermediate arrangement from those of zincblende and wurtzite CdS RQDs. Again, the main difference between MSC-311 and MSC-322 is seen in the fourth peaks. The synchrotron-based results are in excellent

agreement with those obtained with an in-house white X-ray spectrometer (bottom, edited from Supplementary Fig. 6 with a slightly different range for x axis). The high degree of the reproducibility of the PDF work is worthy of notice.

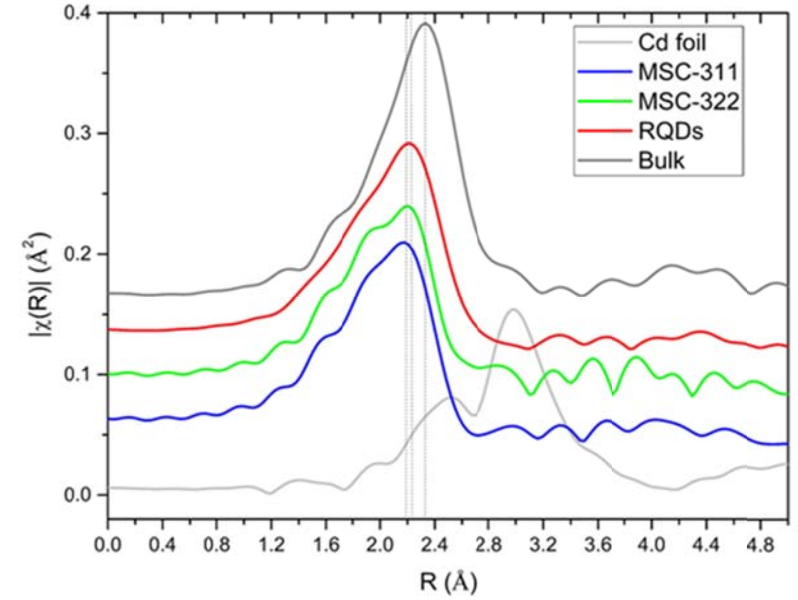

Supplementary Figure 10. Cd K-edge X-ray absorption fine structure (XAFS) measurements was performed in Argonne National Laboratory (beamline 20-BM) for the same two clusters (used for PDF). The spectra were calibrated, averaged, pre-edge background subtracted, and post-edge normalized using Athena program in IFEFFIT software package[19]. The Fourier transformation of the k-weighted EXAFS oscillations, k·χ(k), from k space to R space was performed over a range of 2.2–13.8 $Å^{-1}$ to obtain a radial distribution function. The XAFS results of Cd foil (light grey trace), CdS bulk (grey trace), CdS RQDs (red trace), CdS MSC-311 (blue trace) and CdS MSC-322 (green trace) are showed in the |χ(R)| plots. The peak position represents the nearest-neighbor atom distance around the Cd atoms in a sample. The results show that the nearest-neighbor distance around Cd in the two clusters is very much similar (almost identical), and is slightly different from that of the CdS RQDs and bulk. Again, such similarity indicates a similar local structures for the two clusters.

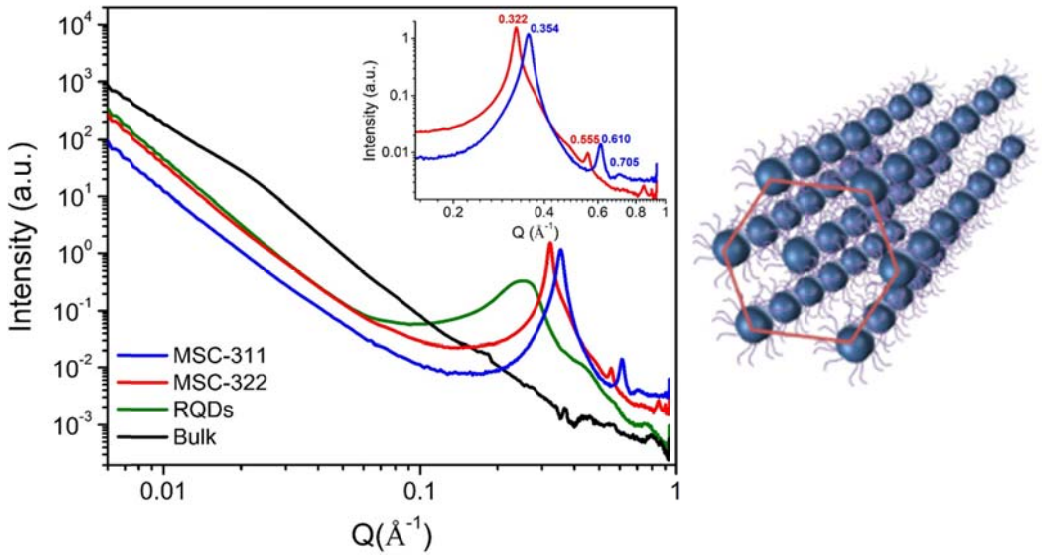

Supplementary Figure 11. (Left) Small angle X-ray scattering (SAXS) performed at Argonne National Laboratory (beamline 12-ID-B) for the same two clusters. Data were collected using X-rays of 14 keV photon energy. The Q-coverage is from 0.006 $Å^{-1}$ up to 0.9 $Å^{-1}$. In addition to powder-form MSC-311 (blue trace) and MSC-322 (red trace), the other two powder-form samples were CdS RQDs (green trace) and bulk CdS (black trace). The powder MSC-311 and MSC-322 samples exhibit a few diffraction peaks in the Q-range of 0.2 – 0.8 $Å^{-1}$ (as indicated in the inset). The q values of those diffraction peaks follow 1 : √3 : 2 for MSC-311 and 1 : √3 for MSC-322, indicating that both powder-form clusters exhibit hexagonal lattices. The particle center-to-center distance of 20.5 Å for MSC-311 and 22.5 Å for MSC-322. (Right) A schematic drawing of the hexagonal pattern (obtained, a real space), which is quite similar to that of self-assembled CdS MSC-322 reported recently[20]. The RQDs (with the size of 22.0 Å (Supplementary Fig. 6)) have a peak at 0.25 $Å^{-1}$, suggesting a random packing with the average particle center-to-center distance of 25.0 Å. If we assume that the two MSCs had the same ligand packing distance (3.0 Å) as that of the RQDs, the sizes of MSC-311 and MSC-322 are then estimated to be 17.5 Å and 19.5 Å, respectively. Therefore, with the similar cluster weight (MS) and the weight ratio of organic ligands to inorganic cores (TGA), MSC-311 and MSC-322 may have slightly different shapes, similar to that reported for TiO_2[17].

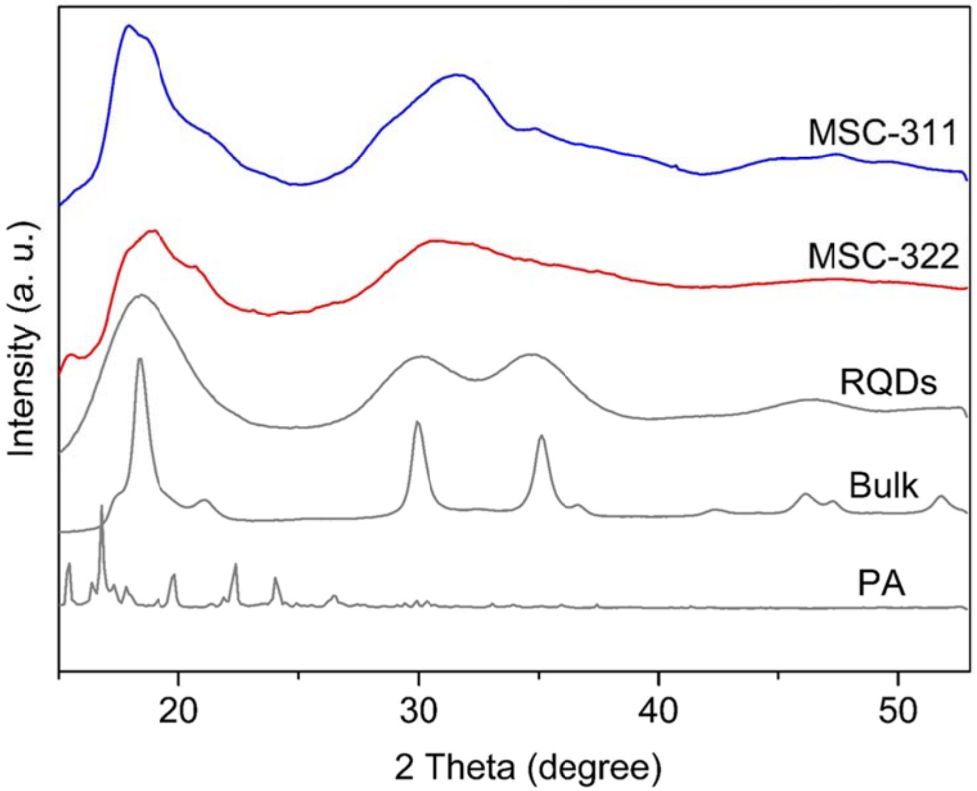

Supplementary Figure 12. Powder WAXS patterns of MSC-311 (blue trace), MSC-322 (red trace), CdS RQDs (gray trace), CdS bulk (gray trace) and phenyl acetic acid (PA, gray trace), arranged from top to bottom. The diffraction peaks of the CdS MSCs and RQDs were broader than those of the CdS bulk. The WAXS patterns of the CdS MSCs and RQDs were different. Also, the WAXS patterns of MSC-311 (blue trace) and MSC-322 (red trace) differed. Such differences were apparent also in the XRD patterns (Supplementary Fig. 13). Thus, the diffraction patterns suggest that MSC-311 and MSC-322 have different crystal structures. The small peak at ~16° for MSC-322 should not be attributed by PA, corresponding to the spacing of ~4 Å (λ = 1.0332 Å). The WAXS measurements were performed at BL16B1 beamline at Shanghai Synchrotron Radiation Facility (SSRF), Shanghai, China, using the X-ray with a wavelength of λ = 1.0332 Å (i.e. with an energy of 12 keV) as the incident beam. With the sample-to-detector distance of 118 mm, powder samples were held and mounted to the sample rack by 3M tape (the background of 3M tape was deducted). The two dimensional (2D) data reduction was done with the Fit 2D software.

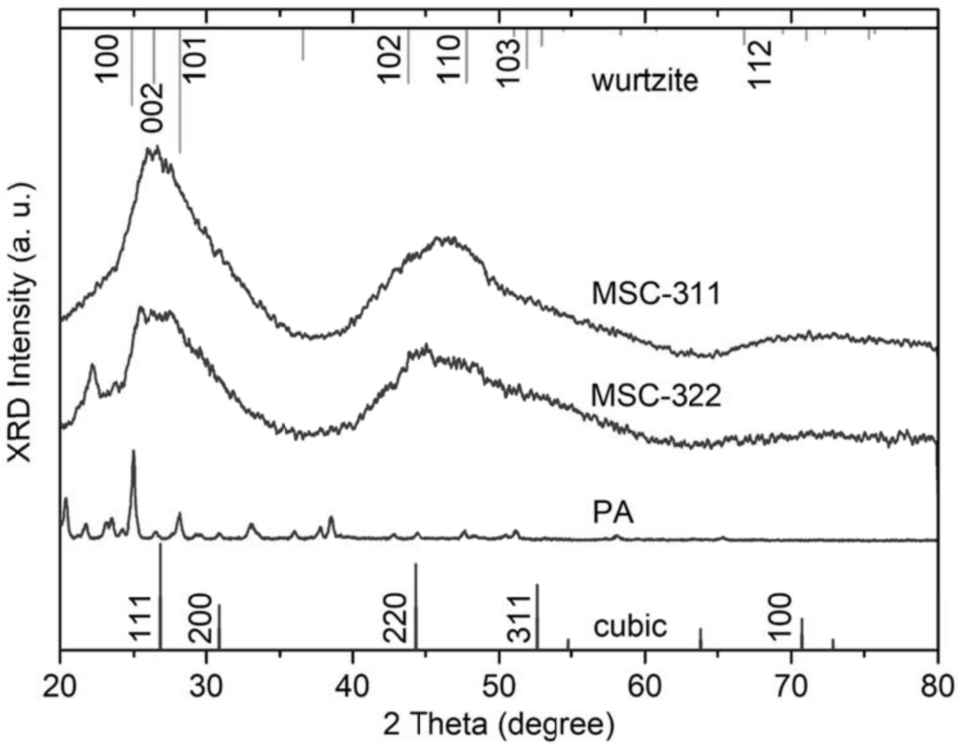

Supplementary Figure 13. Powder X-ray diffraction (XRD) for MSC-311, MSC-322, and PA. The standard diffraction patterns of CdS bulk wurtzite (black vertical lines at the top) and CdS bulk zinc-blende (black vertical lines at the bottom) are included as references. The peak at ~22° of MSC-322 corresponds to the spacing of ~4 Å (λ = 1.5418 Å). The powder XRD patterns were recorded on a Shimadzu X-ray diffractometer 6100 at room temperature, with Cu Kα (λ = 1.5418 Å) radiation in a ϑ-ϑ mode. The generator worked at 40 kV and 40 mA. The data were collected between 10 and 80° in the 2θ mode at a rate of 1° per minute. For the two clusters, the difference presented in our PDF, SAXS, WAXS, and XRD data should not be attributed to the surface ligands (consisting of H, C and O light atoms), but to surface structures due to probable shape differences.

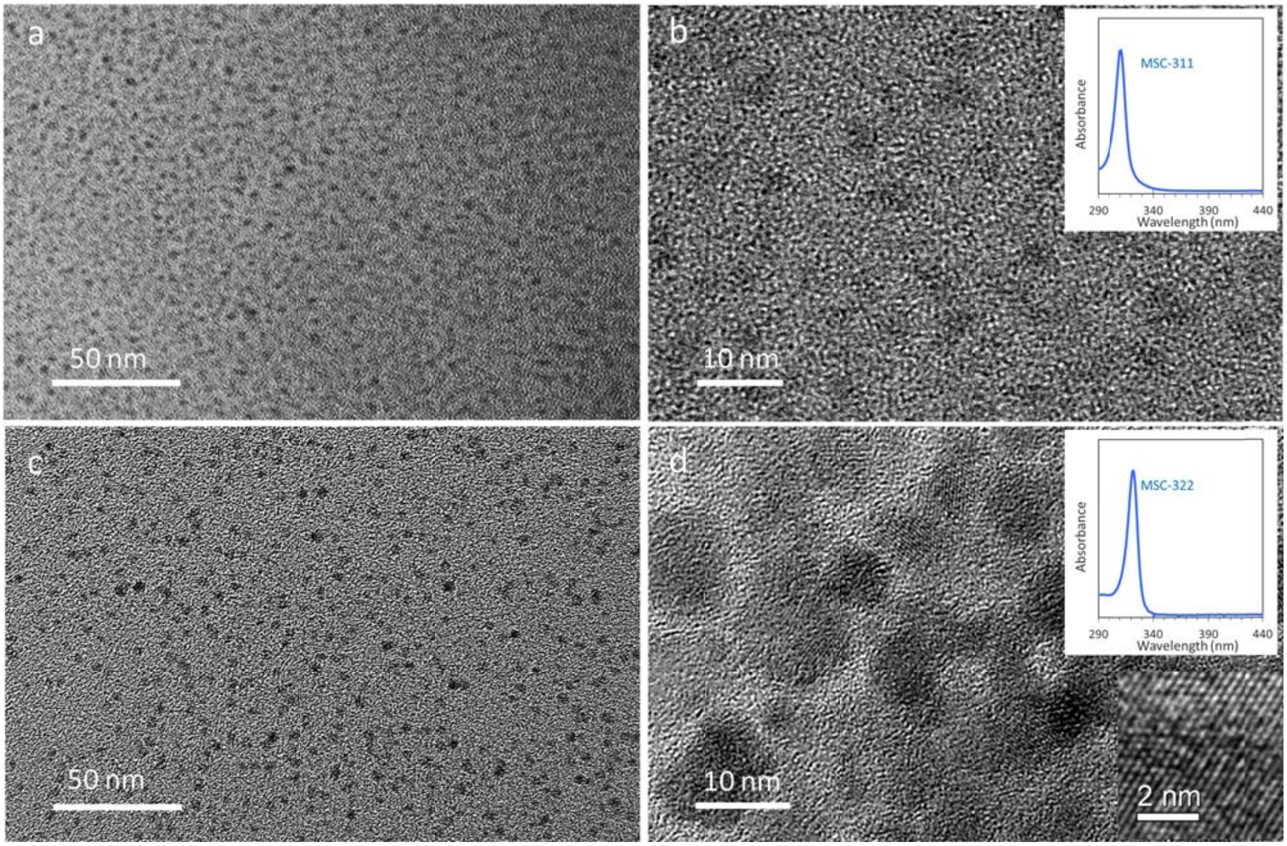

Supplementary Figure 14. TEM images of unpurified MSC-311 (a), purified MSC-311 (b), unpurified MSC-322 (c), and purified MSC-322 (d). The clusters are capped by the oleate ligands. The two insets (b, d) are the corresponding UV-vis absorption spectra. The aggregation seemed to be more significant for the purified samples. The TEM images are not able to represent the actual size of small-size NCs[17,21-22], including MSCs; the underlying reason may be attributed to the changes of clusters such as during TEM sample grid preparation.

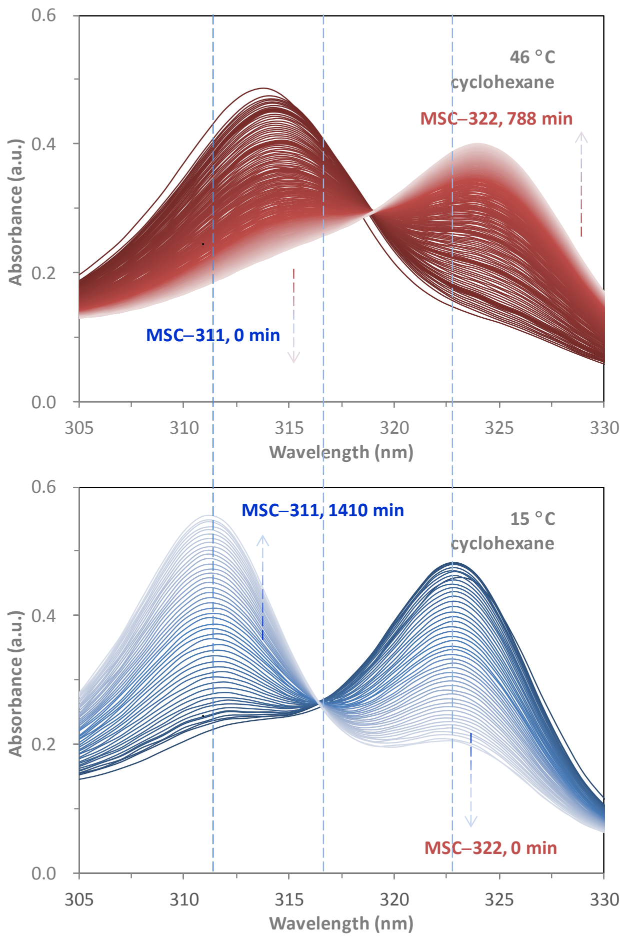

Supplementary Figure 15. Expanded views for the isosbestic points of Figs. 3a and 3b in the range of 305 to 330 nm. (Similar expanded views such as for Figs. 3 and 6 can be found in Supplementary Fig. 26.) The 0 min spectrum of Fig. 3a deviates from the isosbestic point can be attributed to the abrupt change in temperature from room temperature (RT) to 46 °C. The absorption peak positions (including isosbestic points) are affected by temperature, due to the change of molar extinction coefficients with temperature[23]. Fig. 3 shows that there are no other longer wavelength NCs due to the flat baseline (up to 390 nm) involved during the transformation. According to our experimental results (such as shown by figures of Supplementary Figs. 15 and 25, the possible existence of unreacted Cd and S precursors, together with reaction side products and the IP (if there is any), did not seem to affect the isosbestic point feature. Furthermore, the lack of the IPs to MSC-311 conversion can be reasonably attributed to the significantly low IP population. This is due to the fact that the IP

to MSC-311 transformation was revealed to obey a first-order reaction kinetics, with the reaction rate $r = k[\mathrm{IP}]$ (k the rate constant and [IP] the concentration of IPs)[24]. Thus, the concentration of the IP did not affect the conversion rate constant k. Therefore, the transformations studied can be considered as 2-species reactions with the isosbestic point feature presented.

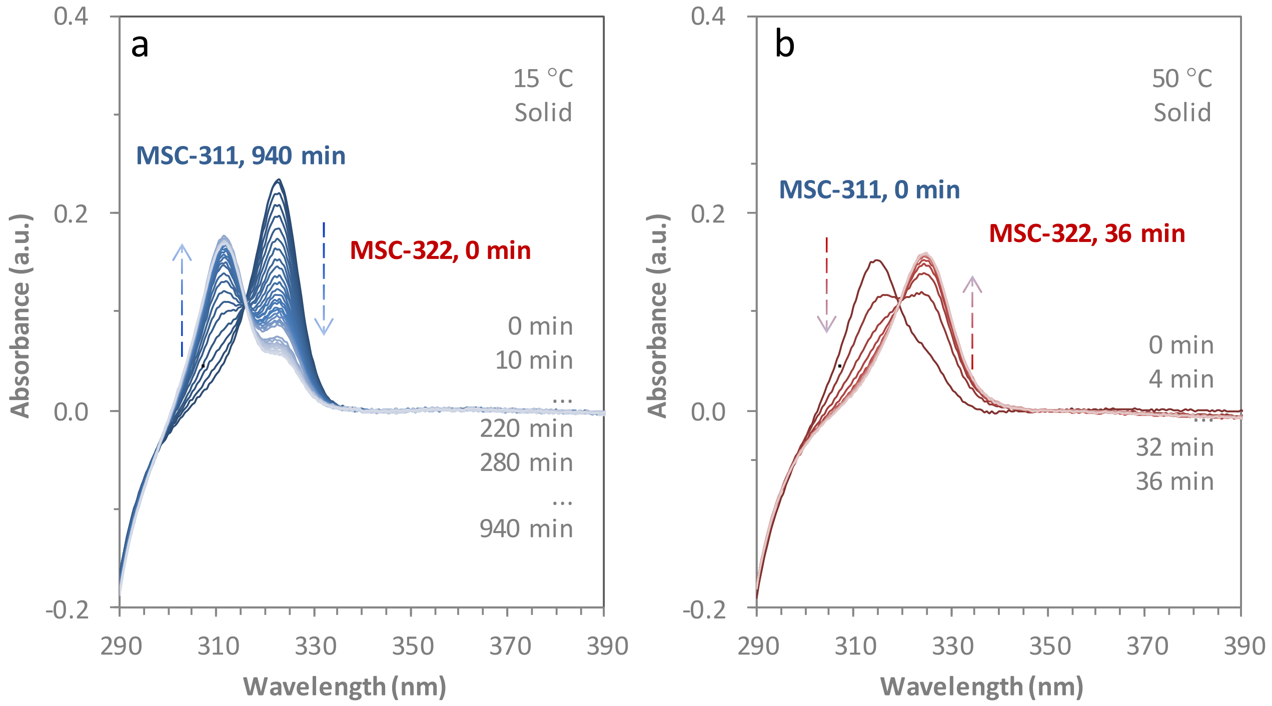

Supplementary Figure 16. *In situ* time-resolved optical absorption spectra collected from a purified solid-state MSC-322 sample placed first at 15 °C (a) and afterwards at 50 °C (b). For the measurement, about 160 mg purified MSC-322 was dispersed in 0.1 mL of toluene. The resulting dispersion was then drop-cast onto the surface of a conventional cuvette, which was then placed into an oven for about 2 hours at 60 °C. Afterwards, the cuvette was placed in the sample holder of our UV-vis spectrometer. When the temperature of the sample stage was set at 15 °C (a), MSC-322 ⇒ MSC-311 occurred. When the sample stage was afterwards set at 50 °C (b), the MSC-311 ⇒ MSC-322 transformation was observed. We will put efforts to investigate solid samples in the future.

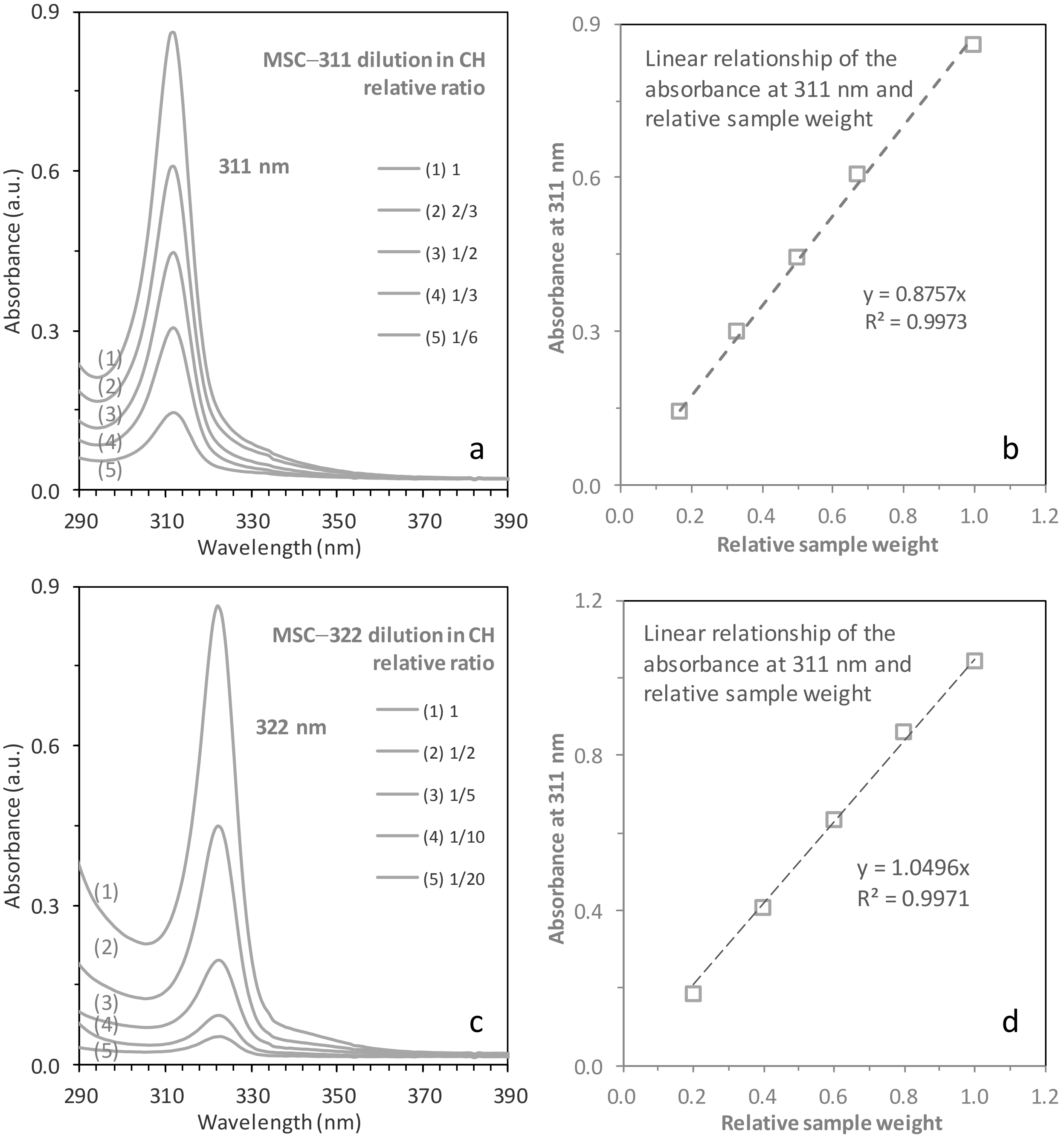
MSC–311 dilution in CH
relative ratio
311 nm
(1) 1
(2) 2/3
(3) 1/2
(4) 1/3
(5) 1/6
Absorbance (a.u.)
Wavelength (nm)
a
Linear relationship of the absorbance at 311 nm and relative sample weight
y = 0.8757x
R² = 0.9973
Absorbance at 311 nm
Relative sample weight
b
MSC–322 dilution in CH
relative ratio
322 nm
(1) 1
(2) 1/2
(3) 1/5
(4) 1/10
(5) 1/20
Absorbance (a.u.)
Wavelength (nm)
c
Linear relationship of the absorbance at 311 nm and relative sample weight
y = 1.0496x
R² = 0.9971
Absorbance at 311 nm
Relative sample weight
d

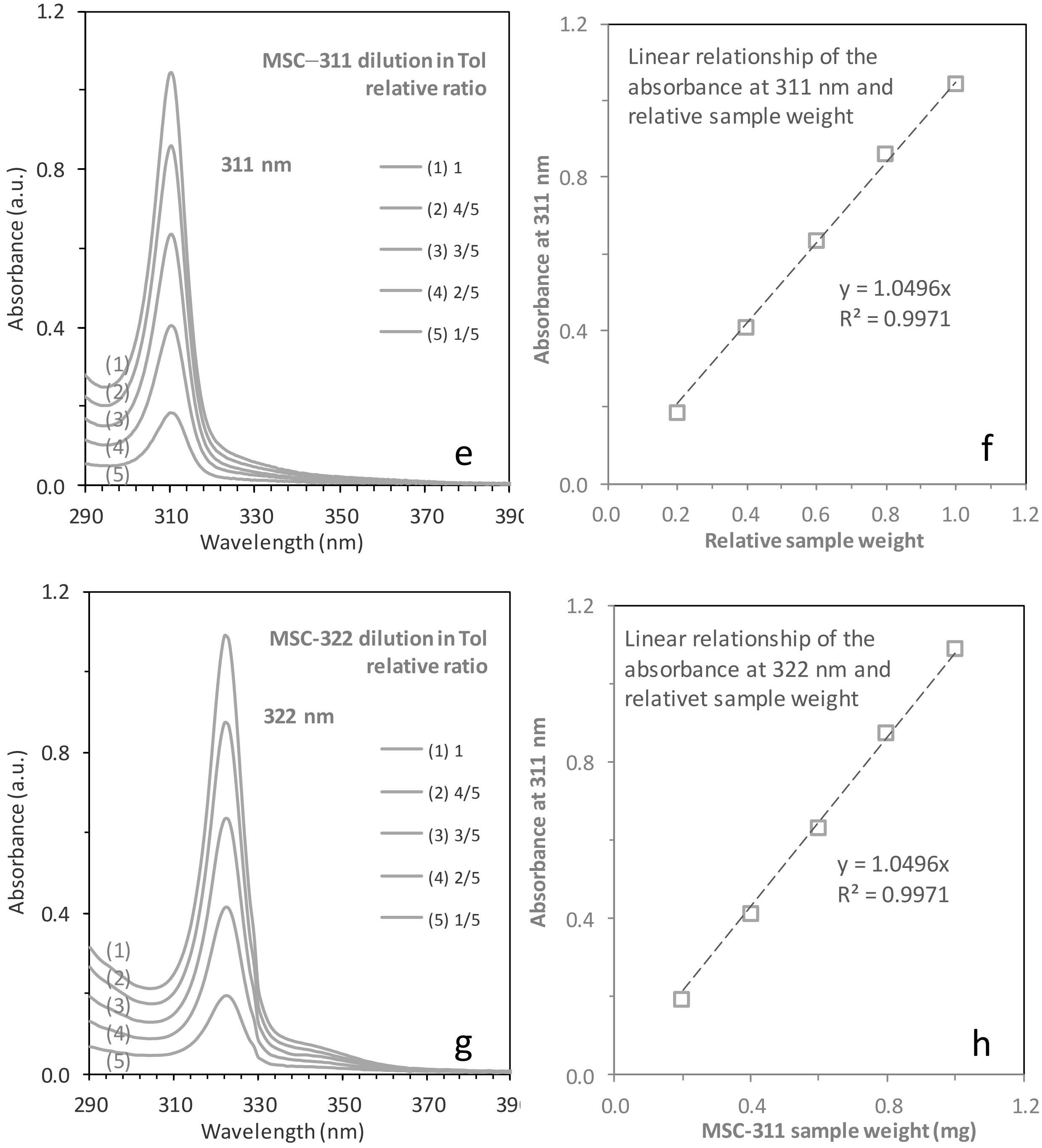

Supplementary Figure 17. Concentration-dependent absorption spectra collected from MSC-311 (a, e) and MSC-322 (c, g) dispersions in cyclohexane (CH, a, c) and in toluene (Tol, e, g), together with the corresponding plots of the absorbance value at 311 nm (b, f) or at 322 nm (d, h) as a function of the relative concentrations (in terms of the degree of dilution). A dilution experiment was performed on Dispersion (1); each of the four dispersions is 3 mL,

with the degree of dilution indicated. The linear relationship obtained between the absorbance at 311 and 322 nm versus concentration indicated that both MSC-311 and MSC-322 were stable (on the experimental time scale), and they did not decompose in the concentration range studied, with a constant extinction coefficient. Interestingly, the slope (which is related to the extinction coefficient) in toluene was larger than that in cyclohexane.

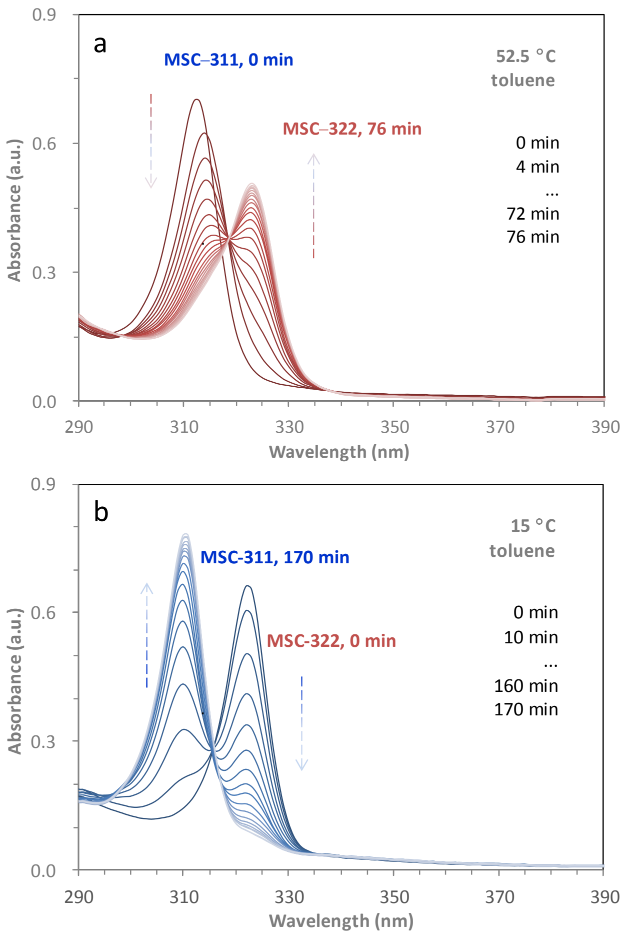

Supplementary Figure 18. *In situ* time-resolved optical absorption spectra collected for the transformation from MSC-311 to MSC-322 at 52.5 °C (a) and for the reverse process of MSC-322 to MSC-311 at 15.0 °C (b). The MSC-311 and MSC-322 samples used were from a preheated sample (~20 mg) that had been stored at –20 °C and 50 °C for 2 days, respectively. The solvent was toluene (3 mL). Again, the 0 min trace (at a relatively high temperature) did not go through the isosbestic point, due to the abrupt change in temperature. See Supplementary Fig. 4. For our kinetic study shown in Fig. 4a, except for the 0 min point (which is away from the first-order reaction behavior and is not considered), the absorbance of MSC-311 was taken according to the MSC-311 peak position of the 4-min spectrum (314 nm). For our kinetic study shown in Fig. 4b, the absorbance of MSC-322 was taken according to the MSC-322 peak position of the 4-min spectrum (323 nm). Similarly, for our kinetic study shown in Fig. 4c, the absorbance of MSC-322 was taken according to the MSC-322 peak position of the 10-min spectrum (321

nm). For our kinetic study shown in Fig. 4d, the absorbance of MSC-311 was taken according to the MSC-311 peak position of the 10-min spectrum (310 nm). By the way, the peak shift during the MSC-311 to MSC-322 transformation was noticed, similar to what observed for the IP-311 to MSC-311 transformation[24] and that reported for PbSe MSCs[25]. To fully understand the present system regarding the peak shift (which seems to be relatively obvious at a relatively high temperature), additional efforts are needed.

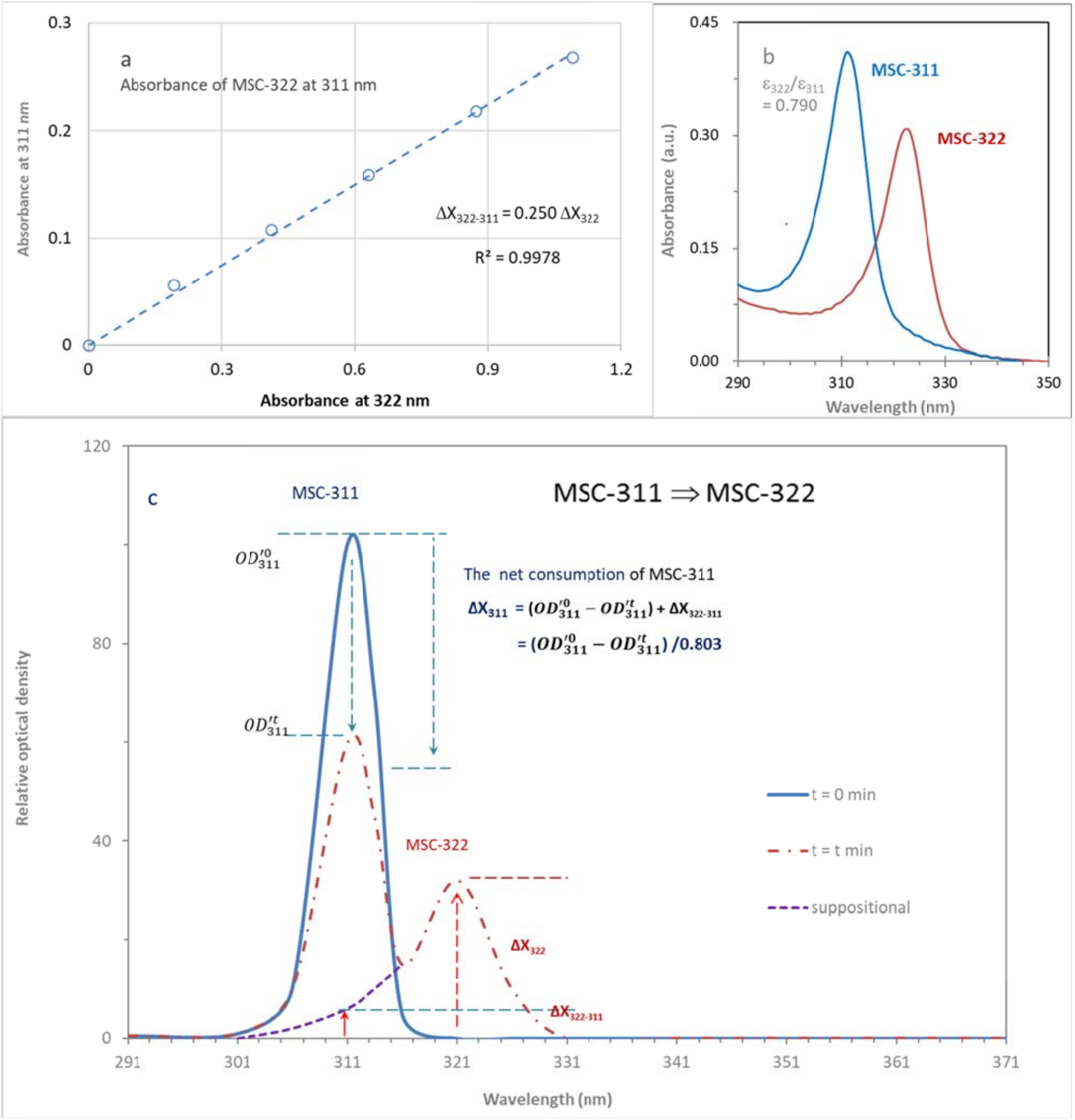

Supplementary Figure 19. Illustration of our mathematical treatment for the temporal absorbance ($\varepsilon C^{t}_{\mathrm{MSC-311}}$) of MSC-311 during MSC-311 ⇒ MSC-322 presented in Fig. 4a. (a) The absorbance at 311 nm (*y* axis) for the various MSC-322 concentrations in toluene (with *x* axis for the absorbance at 322 nm). A linear relationship (dashed line) was obtained (from the dots). (b) Absorption spectra of MSC-311 and MSC-322 collected from one cuvette with

the MSC-311 and MSC-322 transformation in toluene. The two spectra were used to calculate the ratio (0.790) of the molar extinction coefficients of MSC-322 and MSC-311. (c) Numerical treatment accounting for **the off-peak absorbance** of MSC-322 at the peak position of MSC-311. **The net decrease** of absorbance at the peak position of MSC-311 (ΔX_{311}) during MSC-311 ⇒ MSC-322 transformation was thus calculated. For the MSC-311 ⇒ MSC-322 transformation presented in Fig. 4a, the temporal concentration of MSC-311 ($C^{t}_{\mathrm{MSC-311}}$) was considered to be the initial concentration of MSC-311 minuses the net temporal consumption of MSC-311.

The net consumption of MSC-311 was corrected, due to the absorbance contribution of MSC-322 at 311 nm. As shown in Supplementary Fig. 19a, MSC-322 exhibits some absorbance at 311 nm ($\Delta X_{322\text{-}311}$), the strength of which has a linear relationship with the absorbance at 322 nm (ΔX_{322}),

$$\Delta X_{322-311} = 0.250\Delta X_{322} \quad (3)$$

As shown in Supplementary Fig. 19b, the two types of MSCs (in one cuvette and thus with the same concentration) have the relationship of

$$\frac{\varepsilon_{322}}{\varepsilon_{311}} = \frac{OD_{322}}{OD_{311}} = 0.790 \quad (4)$$

(obtained with background subtraction). Thus,

$$\Delta X_{322} = 0.790\ \Delta X_{311} \quad (5)$$

Equation (S1) becomes

$$\Delta X_{322-311} = 0.250 * 0.790\Delta X_{311} \quad (6)$$

As shown in Supplementary Fig. 19c, the net decrease of MSC-311

$$\Delta X_{311} = (OD'^{0}_{311} - OD'^{t}_{311}) + \Delta X_{322-311} = (OD'^{0}_{311} - OD'^{t}_{311}) + 0.250 * 0.790\Delta X_{311} \quad (7)$$

$$\Delta X_{311} = (OD'^{0}_{311} - OD'^{t}_{311})/0.803 \quad (8)$$

Thus, for our kinetic study (Supplementary Fig. 4a), the concentration of MSC-311 equals to the initial concentration of MSC-311 minuses the net consumption of MSC-311,

$$C^{t}_{\mathrm{MSC-311}} = (OD'^{0}_{311} - \Delta X_{311})/\varepsilon_{311} = (1.245OD'^{t}_{311} - 0.245OD'^{0}_{311})/\varepsilon_{311} \quad (9)$$

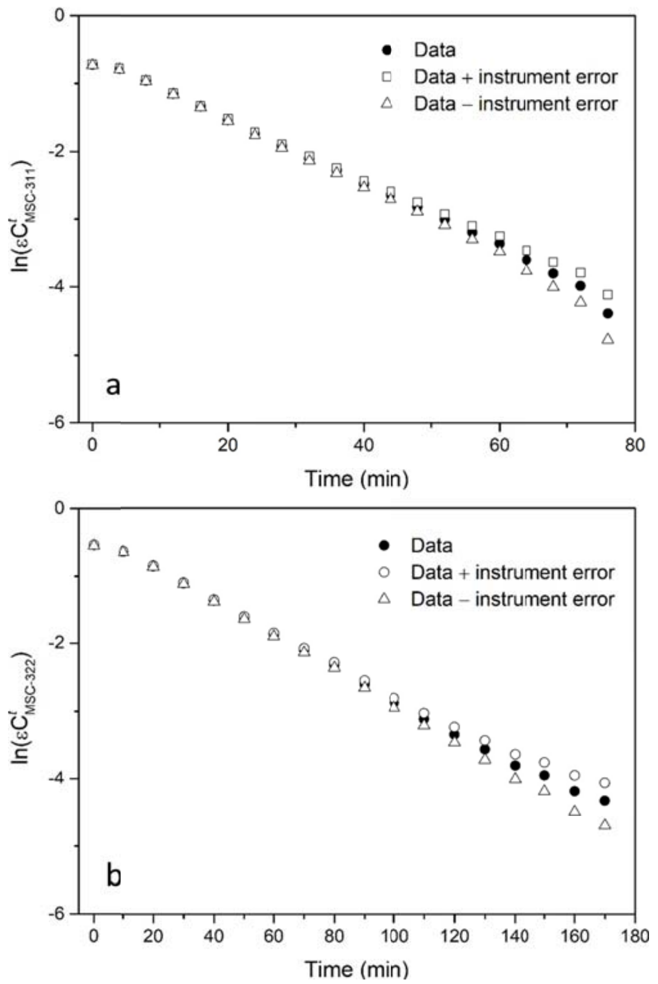

Supplementary Figure 20. The effect of the instrument uncertainty (0.004) associated with the UV-Vis spectrometer (TECHCOMP UV 2310 II) in the kinetic study (using Equation 2) of the MSC-311 to MSC-322 transformation at 52.5 °C (a) as shown in Fig. 4a, and of the MSC-322 to MSC-311 transformation at 15.0 °C (b) as shown in Fig. 4c. After longer times (50 min (a) for MSC-311 to MSC-322 and 80 min (b) for MSC-322 to MSC-311), the concentrations of MSC-311 (a) and MSC-322 (b) were relatively low, where the optical density that we measured was most strongly influenced by the instrument uncertainty (0.004).

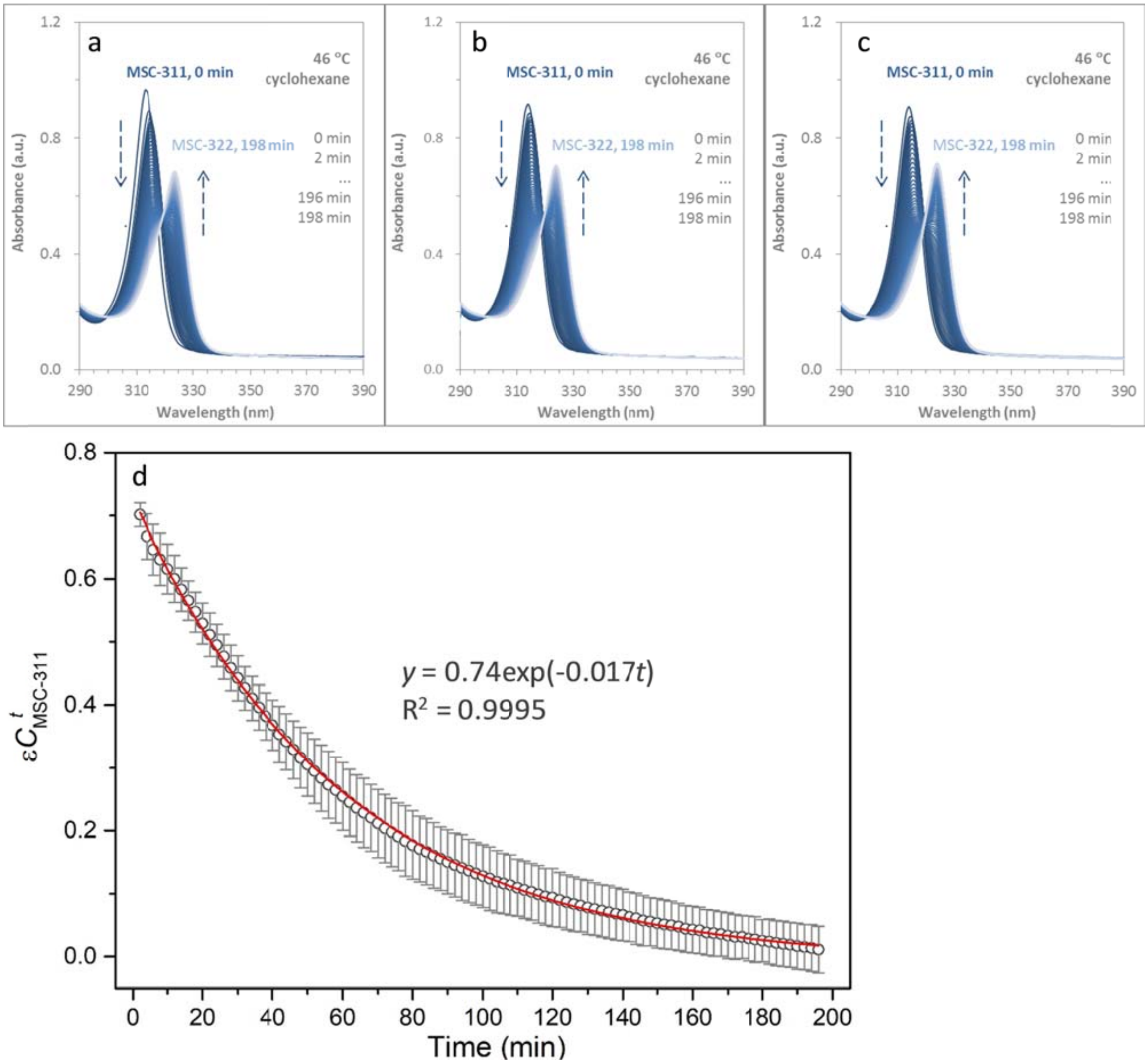

Supplementary Figure 21. Reproducibility in the kinetic study with *in situ* time-resolved absorption spectroscopy. Here we show that reproducible kinetics were obtained for the MSC-311 ⇒ MSC-322 transition. The *in situ* time-dependent absorption spectra were collected from three identical MSC-311 samples (~16 mg dispersed in 3 mL of cyclohexane) every two minutes up to a maximum time of 198 min (a, b, c). The MSC-311 sample was prepared from a preheated sample but stored at –20 °C for days. The time-dependent concentration of MSC-311 (*y* axis, averaged values from the three MSC-311 samples, open symbols), together with the error bars (vertical lines) and the fitted curve (red trace) are

presented (d). The measurement variance obtained was about 6%, suggesting that the reproducibility in our results of the kinetic study is high, for the *in-situ* time-resolved measurements of the MSC-311 to MSC-322 transformation. The rate constant value of 0.017 min^{-1} is smaller than that of 0.047 min^{-1} obtained at 52.5 °C (in toluene, Fig. 4).

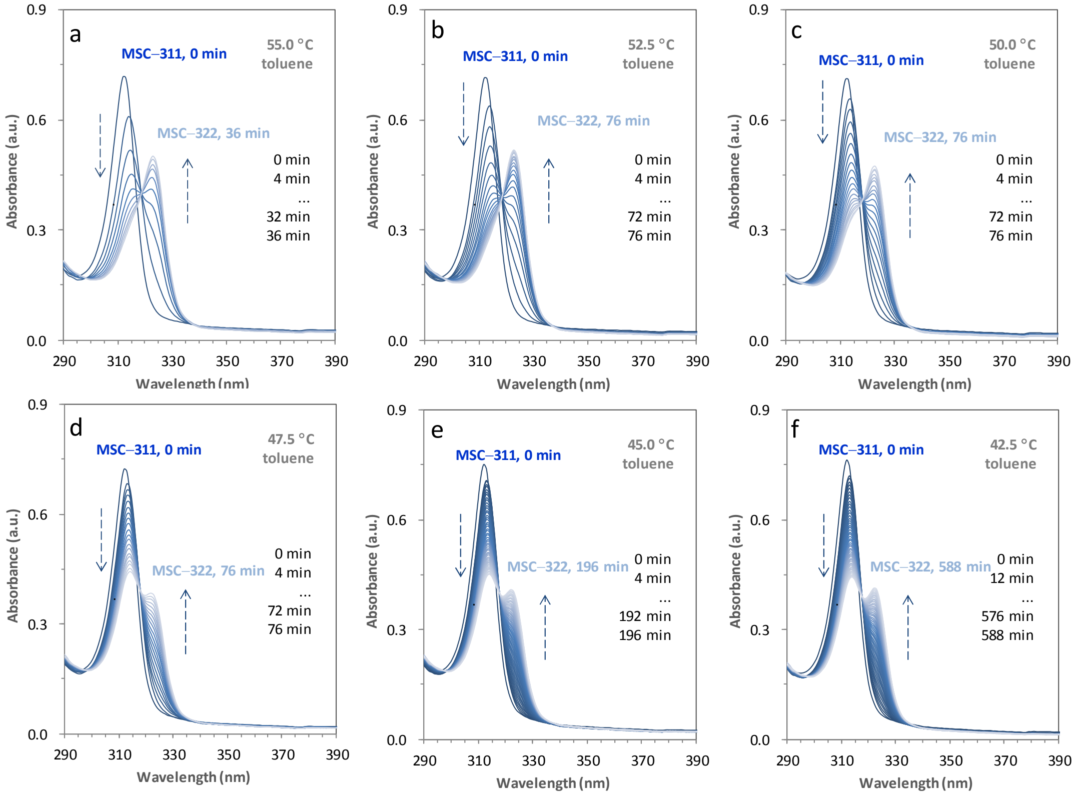

Supplementary Figure 22. *In situ* time-resolved optical absorption spectra collected from six identical MSC-311 dispersions in toluene, which were placed at six different temperatures ranging from 42.5 °C to 55.0 °C (a-f). The dispersion was prepared with MSC-311 (~20 mg) in toluene (3 mL). The spectra were collected with a 4-min time interval, except for the spectra of the sample at 42.5 °C, which was collected with a larger time (12-min) interval because that transformation was much slower for this temperature. The time-resolved absorption spectra are presented with the color change from dark cyan (0 min) to light cyan. It is not easy to study precisely the kinetics of the fast MSC-311 to MSC-322 transformation at 55.0 °C. Our kinetic analysis provided the natural logarithm of the six rate constants, In(k), –2.5 (55 °C, (a)), -3.0 (52.5 °C, (b)), –3.7 (50 °C, (c)), –4.6 (47.5 °C, (d)), –5.4 (45 °C, (e)) and –6.2 (42.5 °C, (f)). The last five rate constants obtained were used to calculate the barrier energy explored in Fig. 5. We would like to point out that for the calculation of a rate constant of the reverse MSC-322 to MSC-311 transformation, we found that it was difficult to obtain a reasonable reproducibility because the transformation proceeded at relatively low temperatures (15 to 25 °C) slowly.

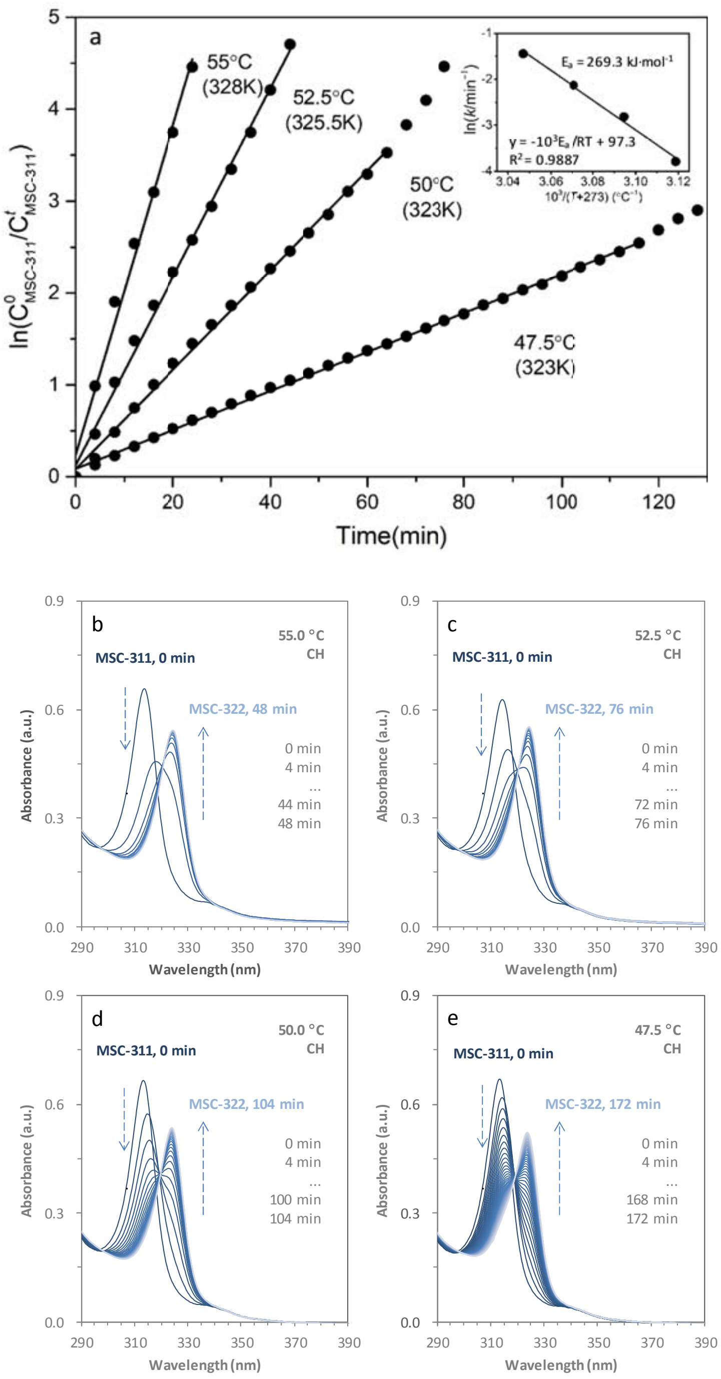

Supplementary Figure 23. First-order reaction kinetic analysis on the MSC-311 ⇒ MSC-322 transformation of four identical dispersions at the four different temperatures (*T*) indicated (from 47.5 °C to 55 °C). The four MSC-311 dispersions in cyclohexane (3 mL) were made from one single MSC-311 sample (~20 mg). The time-dependent $\ln[C^0_{\mathrm{MSC-311}}/C^t_{\mathrm{MSC-311}}]$ (solid circles) was fitted by first-order equations (solid lines, (a)). The slopes of the fitted

lines correspond to the rate constants k (min^{-1}), with In (k) of –1.4 (55.0 °C), –2.1 (52.5 °C), –2.8 (50.0 °C) and –3.8 (47.5 °C). The inset (a) shows an Arrhenius plot, with a y axis of ln(k) and an x axis of 1/T; thus, the slope of the fitted line is E_a/R, where R the ideal gas constant and with a barrier energy, E_a = 269.3 kJ*mol^{-1}. *In situ* time-resolved optical absorption spectra data are shown as below from 47.5 °C to 55.0 °C (b-e). By a side note, we also performed thermodynamic analysis, as shown by Supplementary Figure 24. For the MSC-322 $\Rightarrow$ MSC-311 transformation, we tried to accelerate with CH_3OH, as shown by Supplementary Figure 25.

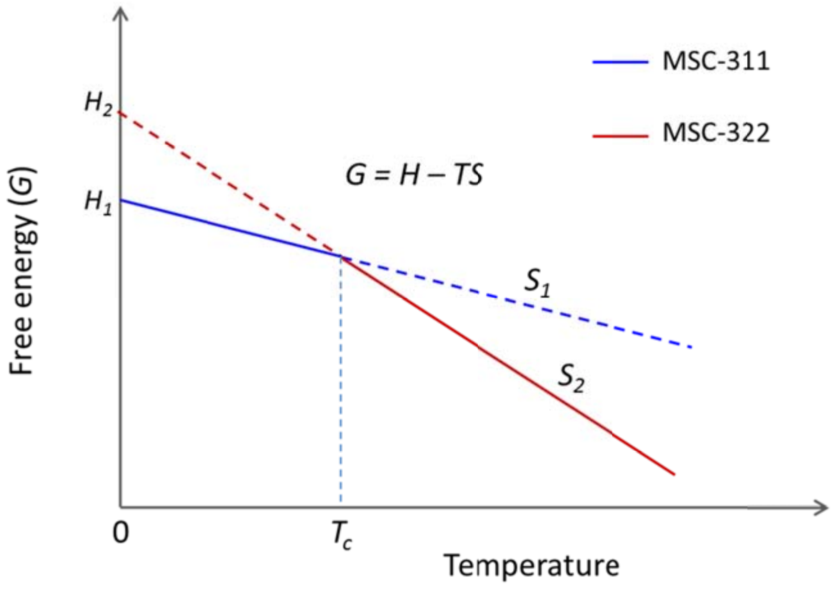

Supplementary Figure 24. Schematic drawing for our thermodynamic analysis of the reconstructive solid-solid transformation which can be effectively controlled by temperature. The abscissa is the temperature (T) scale, and the ordinate is the free energy $G = H - TS$, comprised of the enthalpy H and entropy S. Depicted are the temperature-dependent free energies ($G = H - TS$) of MSC-311 (blue line) and MSC-322 (burgundy line). The slopes of the two lines represent the negative entropy of MSC-311 (S_1) and MSC-322 (S_2) and the y-intercepts of the two lines comprise the enthalpies of MSC-311 (H_1) and MSC-322 (H_2). The transformation is driven by a balance between changes in entropy (due to changes in the atomic vibrations) and enthalpy (due to changes in bonding). To the best of our knowledge the reversible solid-solid transformation between MSC-311 and MSC-322 is the first such thermally-induced isomerization process of colloidal NCs ever reported. Obtained is the relation of $H_2 > H_1$ and $S_2 > S_1$. When a temperature is above a critical point (T_c), the forward MSC-311 to MSC-322 transformation is promoted thermodynamically, with a relatively low G for MSC-322 and $\Delta H = H_2 - H_1 > 0$ and $\Delta S = S_2 - S_1 > 0$. When a temperature is below this critical point (T_c), the reverse MSC-322 to MSC-311 transformation is driven thermodynamically, with a relatively low G for MSC-311 and $\Delta H = H_1 - H_2 < 0$ and $\Delta S = S_1 - S_2 < 0$. Our conclusion is that the forward MSC-311 to MSC-322 transformation at $T > T_c$ is mainly ΔS (entropy) driven, while the reverse MSC-322 to MSC-311 transformation at $T > T_c$

is mainly ΔH (enthalpy) driven. Thus, both transformation are dictated by changes in the balance between entropy (due to atomic vibrations) and enthalpy (due to bonding).

Lastly, we would like to provide a side note about our additional thoughts for solid-solid transformations of large colloidal particles reported[26,27]. When a domain consists of a small number of particles (such as ~70 instead of ~1700), we anticipate that an "isomerization-like" single nucleation even with first-order unimolecular reaction kinetics may take place in one step, without intermediates being involved, for solid-solid transformations with large colloidal particles of ~0.7 μm in size.

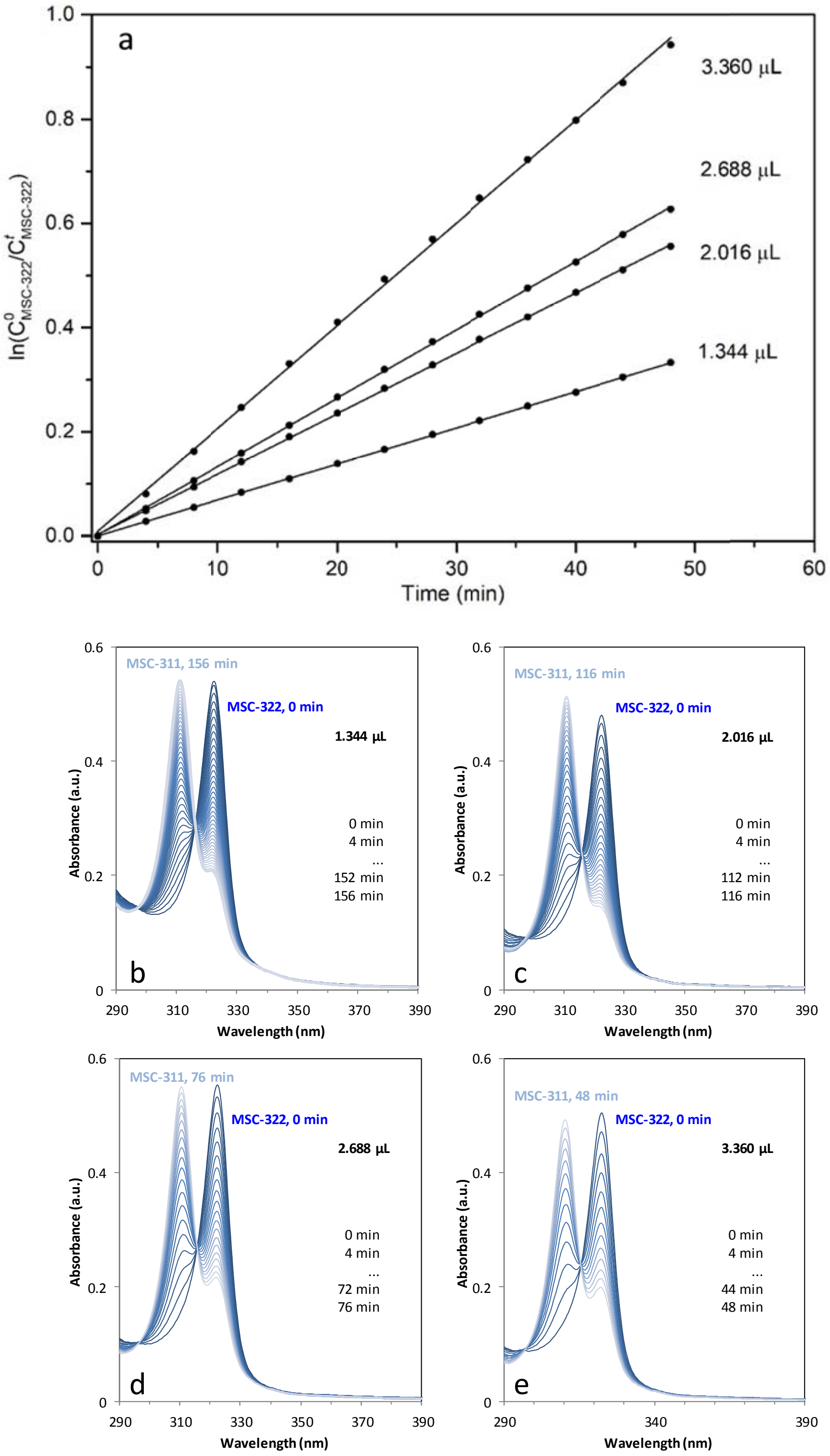

Supplementary Figure 25. A small amount of MeOH was employed as an additive to accerlate the MSC-322 to MSC-311 transformation at 25 °C. (a) Our preliminary kinetic study

for the CH_3OH-induced MSC-322 $\Rightarrow$ MSC-311 transformation. (b-e) Absorption spectra collected. One dispersion was prepared and divided into four identical portions (with ~20 mg MSC-322 sample each in toluene (3 mL). CH_3OH (1.344 μL, 2.016 μL, 2.688 μL and 3.360 μL) was added into each portion. The time-dependent $\ln(C^{0}_{\mathrm{MSC-322}}/C^{t}_{\mathrm{MSC-322}})$ (solid circles) was fitted by first-order equations (solid lines, top). The slopes of the fitted lines correspond to the rate constants k (min^{-1}), with ln(k) of –5.1 (1.344 μL), –4.5 (2.016 μL), –4.4 (2.688 μL) and –3.9 (3.360 μL). Here, CH_3OH acted as a catalyst to accelerate the MSC-322 to MSC-311 transformation, similar to that reported for the IP to MSC-311 transformation at room temperature[1]. Accordingly, it seems reasonable to conclude that the surface rearragement[11,17,23] can play a crtical role for the optical transformation detected in the present study.

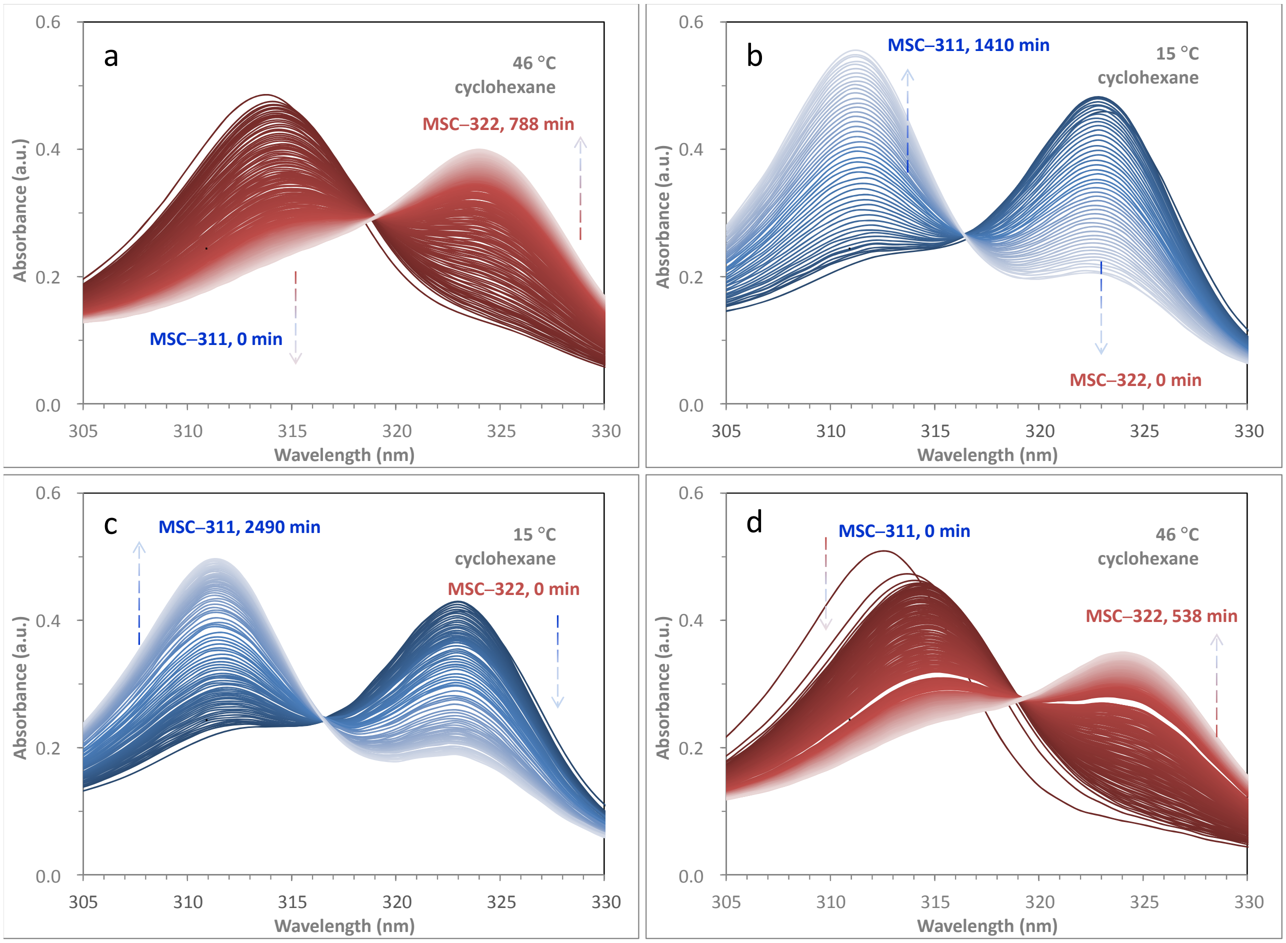

Supplementary Figure 26. Expanded views of Figs. 3a-d (a-d) (with the wavelength range of 25 nm from 305 to 330 nm) to show the isosbestic point feature. The isosbestic point presents in both the forward and reverse transformations at different temperatures. By the way, we did mention in Supplementary Fig. 15 caption "The 0 min spectrum (top) deviates from the isosbestic point can be attributed to the abrupt change in temperature from room temperature (RT) to 46 °C".

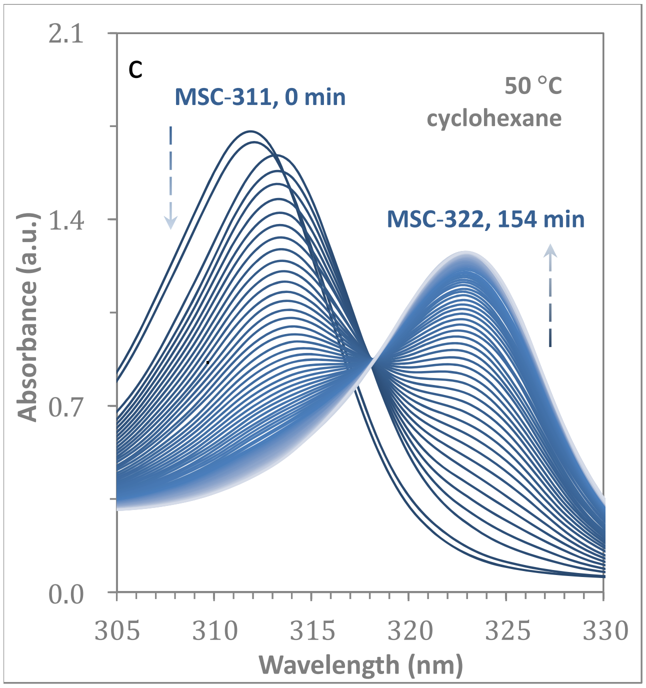

Supplementary Figure 27. Expanded views of Figs. 6c (c) (with the wavelength range of 25 nm from 305 to 330 nm) to show the isosbestic point feature. The isosbestic point presents in different solvents (Tol, CH).

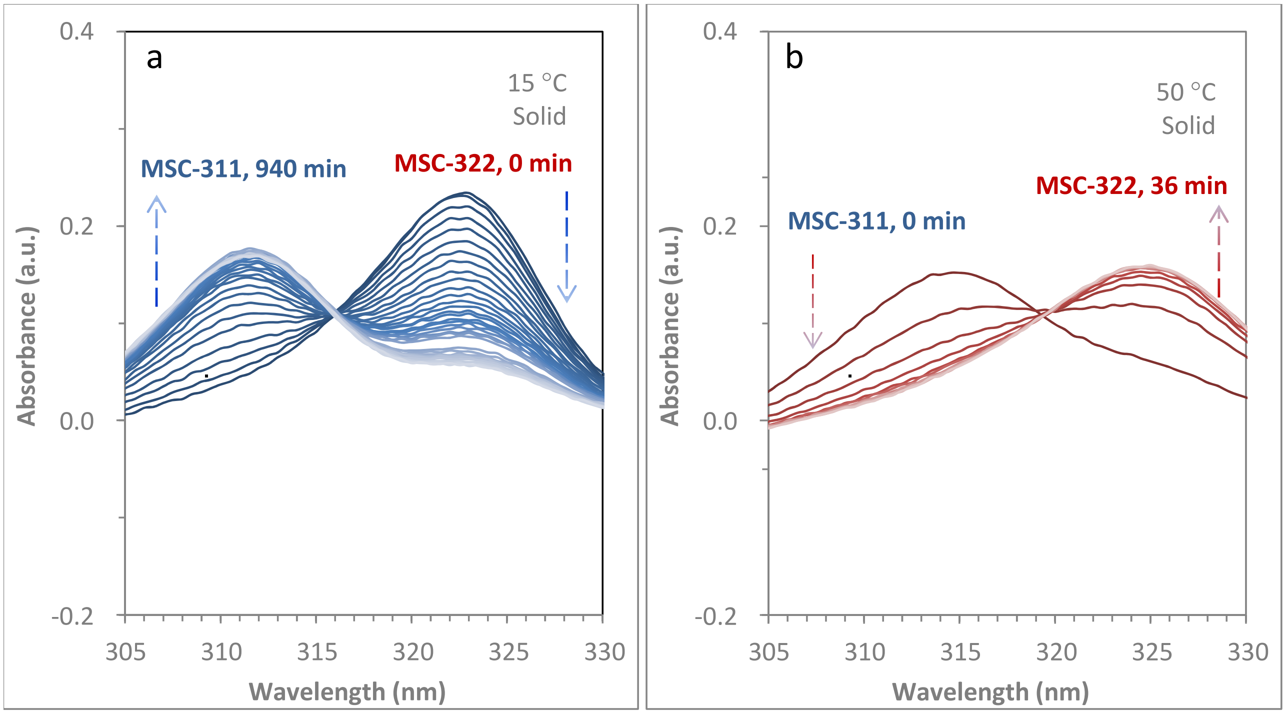

Supplementary Figure 28. Supplementary Figs. 16a-b (a-b) (with the wavelength range of 25 nm from 305 to 330 nm) to show the isosbestic point feature. The isosbestic point presents in both the forward and reverse transformations at different temperatures, and with different material state (solid and dispersion).

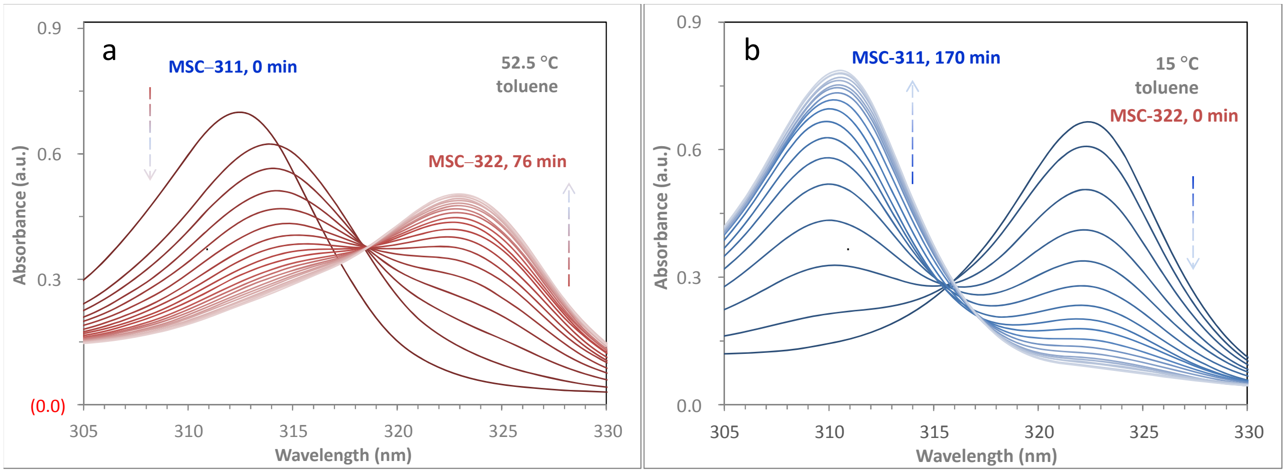

Supplementary Figure 29. Expanded views of Supplementary Figs. 16a-b (a-b) (with the wavelength range of 25 nm from 305 to 330 nm) to show the isosbestic point feature. The isosbestic point presents in both the forward and reverse transformations at different temperatures.

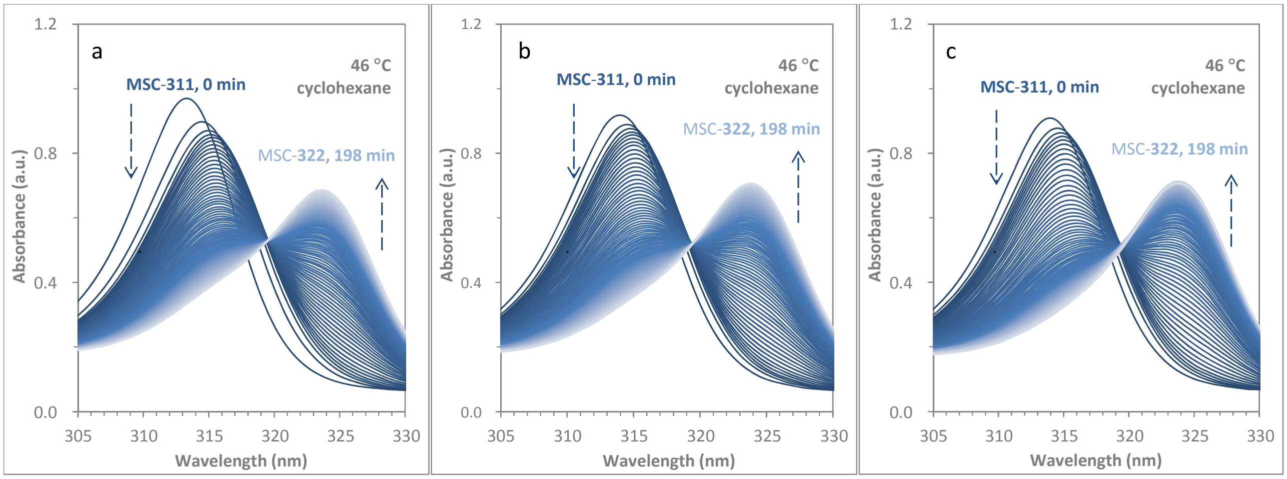

Supplementary Figure 30. Expanded views of Supplementary Figs. 21a-c (a-c) (with the wavelength range of 25 nm from 305 to 330 nm) to show the isosbestic point feature. The isosbestic point repeated well in the forward transformations.

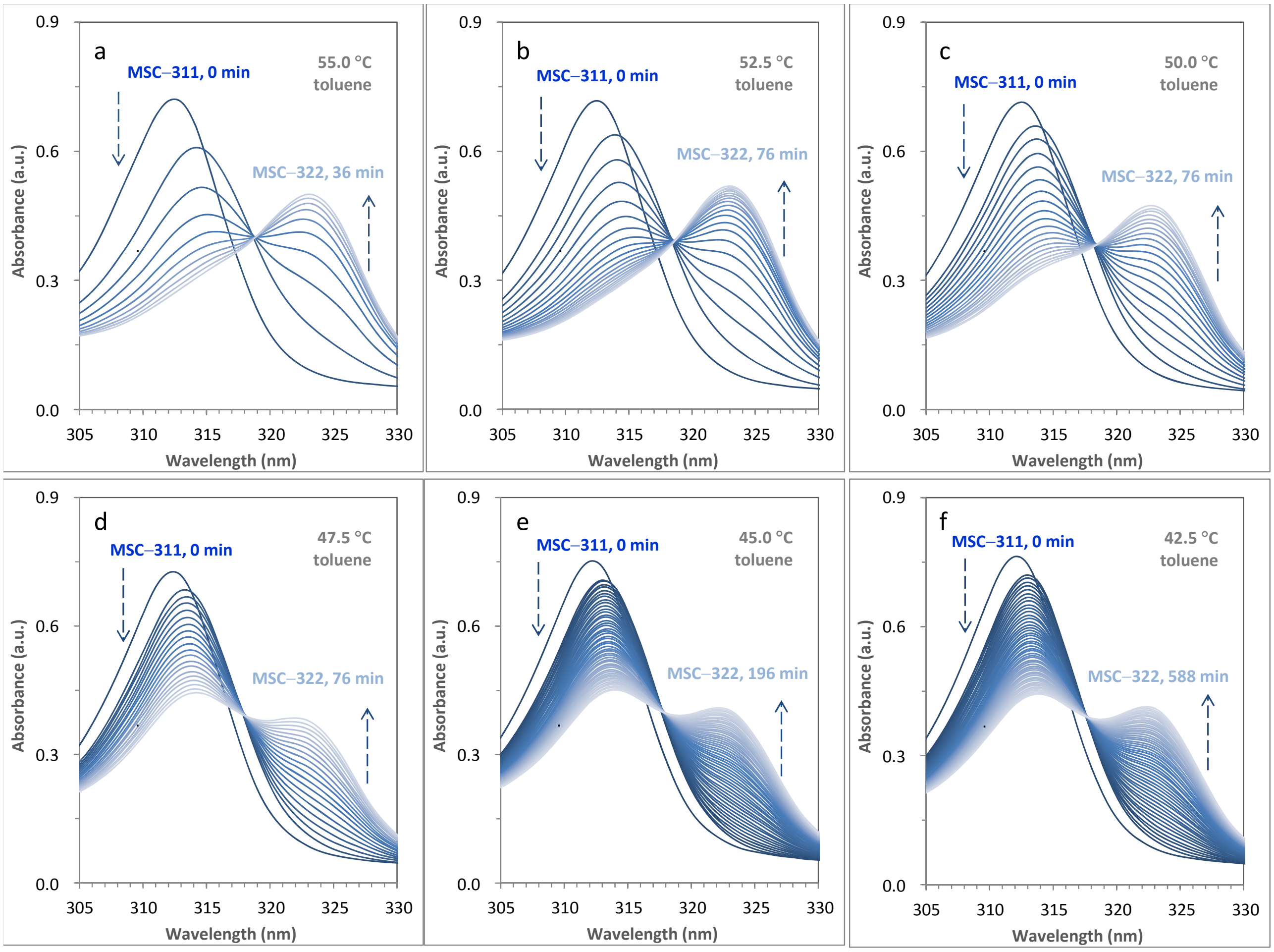

Supplementary Figure 31. Expanded views of Supplementary Figs. 22a-f (a-f) (with the wavelength range of 25 nm from 305 to 330 nm) to show the isosbestic point feature. The isosbestic point presents at the different temperatures.

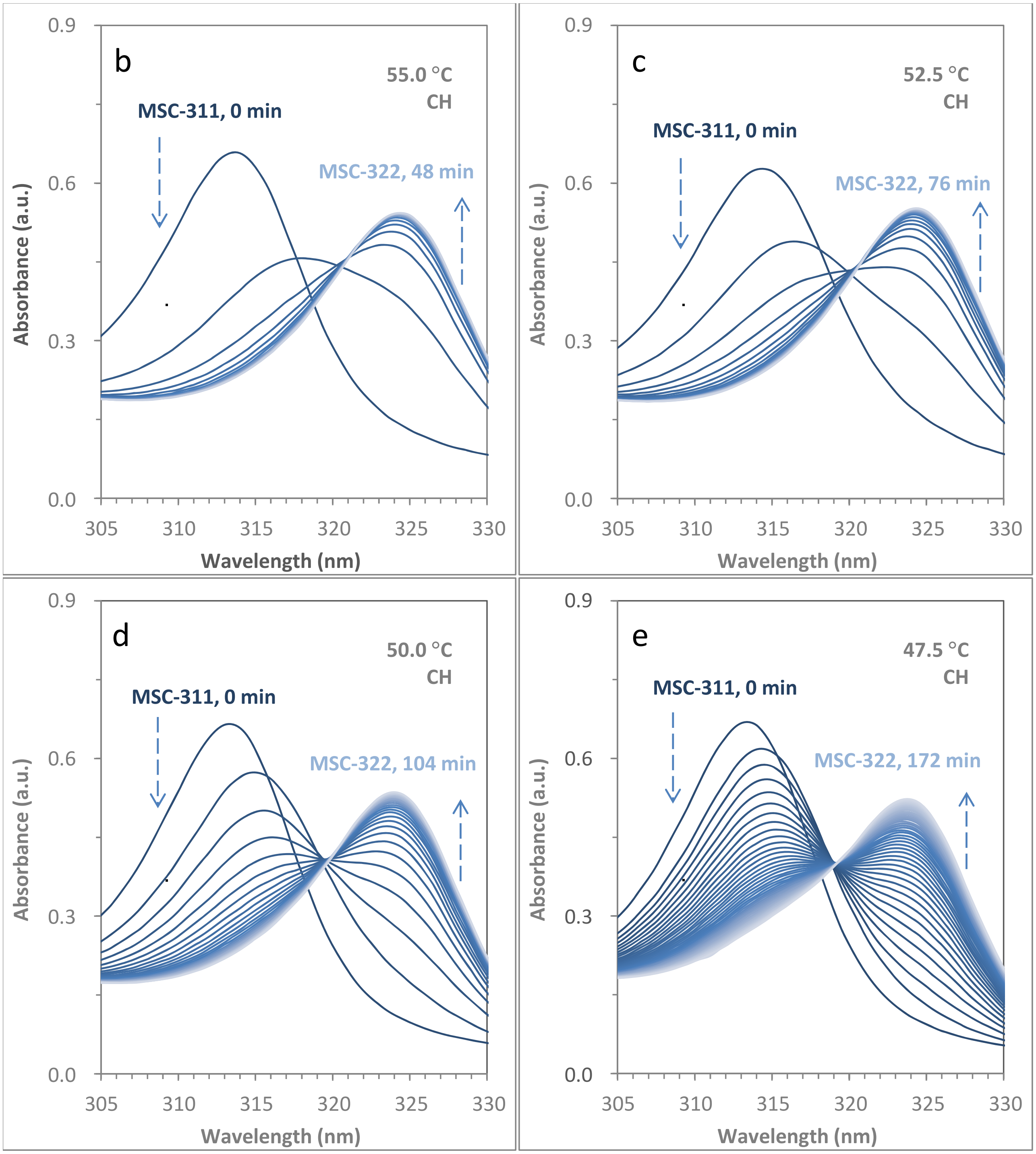

Supplementary Figure 32. Expanded views of Supplementary Figs. 23b (b), 23c (c), 23d (d), 23e (e) (with the wavelength range of 25 nm from 305 to 330 nm) to show the isosbestic point feature. The isosbestic point presents at different temperatures, in different solvents (Tol, CH, and mixtures of Tol and MeOH).

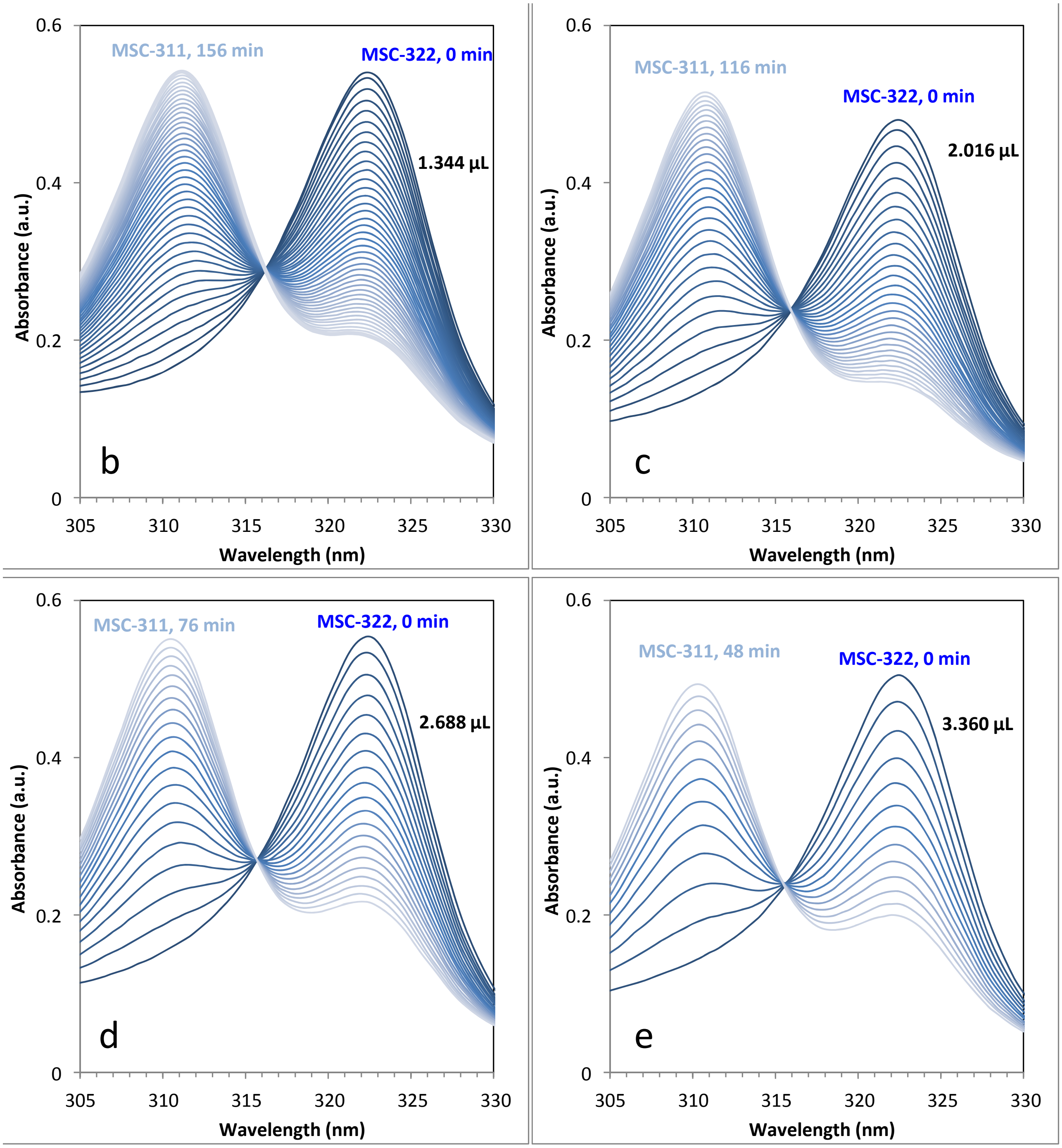

Supplementary Figure 33. Expanded views of Supplementary Figs. 25b (b), 25c (c), 25d (d), 25e (e) (with the wavelength range from 305 to 330 nm) to show the isosbestic point feature. The isosbestic point presents in both the forward and reverse transformations at different temperatures, in different solvents (Tol, CH, and mixtures of Tol and MeOH), and with different material state (solid and dispersion). The isosbestic point feature should not be a coincidence.

Supplementary References

1. Egami, T., & Billinge, S. J. L. *Underneath the Bragg peaks. Structural analysis of complex materials*. (Pergamon press, 2003).
2. Dinnebier, R. E., & Billinge, S. J. L. *Powder diffraction: theory and practice*. (RSC Publishing, 2008).
3. Masadeh, A. S., Božin, E. S., Farrow, C. L., Paglia, G., Juhas, P., & Billinge, S. J. L. Quantitative size-dependent structure and strain determination of CdSe nanoparticles using atomic pair distribution function analysis. *Phys. Rev. B* **76,** 115413 (2007).
4. Shi, C., Redmond, E. L., Mazaheripour, A., Juhas, P., Fuller, T. F. & Billinge, S. J. L. Evidence for anomalous bond softening and disorder below 2 nm diameter in carbon-supported platinum nanoparticles from the temperature-dependent peak width of the atomic pair distribution function. *J. Phys. Chem. C* **117,** 7226– 7230 (2013).
5. Yang, X. H. *et al.* Confirmation of disordered structure of ultrasmall CdSe nanoparticles from X-ray atomic pair distribution function analysis. *Phys. Chem. Chem. Phys.* **15,** 8480–8486 (2013).
6. Farrow, C. L. *et al*. PDFfit2 and PDFgui: computer programs for studying nanostructure in crystals. *J. Phys. Condens. Matter.* **19,** 335219 (2007).
7. Mendoza, L. G., Terban M. W., Billinge, S. J. L. & Inesta, M. M. Modelling and validation of particle size distributions of supported nanoparticles using the pair distribution function technique. *J. Appl. Cryst.* **50,** 741-748 (2017).
8. Gilbert, B. Finite size effects on the real-space pair distribution function of nanoparticles. *J. Appl. Cryst.* **41,** 554-562 (2008).
9. Tolbert, S. H. & Alivisatos, A. P. Size dependence of a first order solid-solid phase transition: the wurtzite to rock salt transformation in CdSe nanocrystals. *Science* **265,** 373–376 (1994).
10. Tolbert, S. H. & Alivisatos, A. P. The wurtzite to rock salt structural transformation in CdSe nanocrystals under high pressure. *J. Chem. Phys.* **102,** 4642–4656 (1995).
11. Chen, C. C., Herhold A. B., Johnson, C. S. & Alivisatos, A. P. Size dependence of structural metastability in semiconductor nanocrystals. *Science* **276,** 398–401 (1997).
12. Jacobs, K., Zaziski, D., Erik, C., Herhold, A. B. & Alivisatos, A. P. Activation volumes for solid-solid transformations in nanocrystals. *Science* **293,** 1803–1806 (2001).
13. Herron, N., Calabrese, J. C., Farneth, W. E. & Wang, Y. Crystal structure and optical properties of $Cd_{32}S_{14}(SC_6H_5)_{36}{\cdot}DMF_4$, a cluster with a 15 angstrom CdS core. *Science* **259,** 1426–1428 (1993).
14. Kim, B. H. *et al.* Sizing by weighing: characterizing sizes of ultrasmall-sized iron oxide nanocrystals using MALDI-TOF mass spectrometry. *J. Am. Chem. Soc.* **135,** 2407–2410 (2013).
15. Kasuya, A. *et al.* Ultra-stable nanoparticles of CdSe revealed from mass spectrometry. *Nat. Mater.* **3,** 99–102 (2004).
16. Beecher, A. N. *et al.* Atomic structures and gram scale synthesis of three tetrahedral quantum dots. *J. Am. Chem. Soc.* **136,** 10645–10653 (2014).
17. Hua, X., Liu, Z., Bruce, P. G. & Grey, C. P. The morphology of TiO_2 (B) nanoparticles. *J. Am. Chem. Soc.* **137,** 13612−13623 (2015).

18. Toby, B. H. & Dreele, R. B. V. GSAS-II: the genesis of a modern open-source all purpose crystallography software package. *J. Appl. Cryst*. **46**, 544–549 (2013).
19. Newville. M. IFEFFIT : interactive XAFS analysis and FEFF fitting. *J. Synchrotron Rad.* **8,** 322–324 (2001).
20. Nevers, D. R. et al. Mesophase formation stabilizes high-purity magic-sized clusters. *J. Am. Chem. Soc.* **140,** 3652–3662 (2018).
21. Zanella, M., Abbasi, A. Z., Schaper, A. K. & Parak, W. J. Discontinuous growth of II-VI semiconductor nanocrystals from different materials. *J. Phys. Chem. C* **114,** 6205–6215 (2010).
22. Xie, L. *et al*. Characterization of indium phosphide quantum dot growth intermediates using MALDI-TOF mass spectrometry. *J. Am. Chem. Soc.* **138,** 13469–13472 (2016).
23. Vossmeyer, T. *et al*. CdS nanoclusters: synthesis, characterization, size dependent oscillator strength, temperature shift of the excitonic transition energy, and reversible absorbance shift. *J. Phys. Chem.* **98,** 7665–7673 (1994).
24. Zhu, T. *et al.* Two-step nucleation of CdS magic-size nanocluster MSC–311. *Chem. Mater.* **29,** 5727–5735 (2017).
25. Yu, K., Ouyang, J. & Leek, D. M. In-situ observation of nucleation and growth of PbSe magic-sized nanoclusters and regular nanocrystals. *Small* **7,** 2250–2262 (2011).
26. Li, B., Zhou, D. & Han, Y. Assembly and phase transitions of colloidal crystals. *Nat. Rev. Mater.* **1,** 15011 (2016).
27. Peng, Y., Li, W., Wang, F., Still, T., Yodh, A. G. & Han, Y. Diffusive and martensitic nucleation kinetics in solid-solid transitions of colloidal crystals. *Nat. Commun.* **8,** 14978 (2017).
